# ﻿Two new species of Sminthurididae (Hexapoda, Collembola, Symphypleona) from Brazil with notes on *Denisiella* Folsom & Mills and Sphaeridia Linnaniemi

**DOI:** 10.3897/zookeys.1173.106855

**Published:** 2023-07-31

**Authors:** Gleyce da Silva Medeiros, Clécio Danilo Dias da Silva, Josemária Silva de França, Nerivânia Nunes Godeiro, Bruno Cavalcante Bellini

**Affiliations:** 1 Department of Botany and Zoology, Biosciences Center, Federal University of Rio Grande do Norte (UFRN), Highway BR-101, Lagoa Nova, Campus Universitário, Natal 59072-970, RN, Brazil Federal University of Rio Grande do Norte (UFRN) Natal Brazil; 2 Shanghai Natural History Museum, Shanghai 200041, China Shanghai Natural History Museum Shanghai China

**Keywords:** Chaetotaxy, groups of species, Neotropical Region, Sminthuridida, Sminthuridoidea, taxonomy

## Abstract

Two new species of Sminthurididae, *Sphaeridiapiauiensis* Medeiros & Bellini, **sp. nov.** and *Denisiellapiracurucaensis* Silva, Medeiros & Bellini, **sp. nov.** from Piaui state, Brazil, are herein described and illustrated. *Sphaeridiapiauiensis***sp. nov.** resembles species of the *irmleri* group, like *S.irmleri* Bretfeld & Gauer, *S.fibulifera* Bretfeld & Gauer, and *S.peruensis* Bretfeld & Schulz, by its complex male ventral tube without asymmetrical structures or medial process. However, it differs from them by the combination of the male tibiotarsus III with a leaf-shaped IIpe chaeta and a regular IIIpi chaeta, ventral tube with 1+1 chaetae, and the absence cuticular hooks on the furca. *Denisiellapiracurucaensis***sp. nov.** resembles its congeners without the nasal organ, especially *D.colombiana* Ospina & Palacios-Vargas, by the presence of spiniform chaetae at least on the second antennal segment of the females, four serrated spines on tibiotarsus III, and the ventral dens chaetotaxy, but *D.piracurucaensis***sp. nov.** differs from the latter especially by the presence of 8+8 eyes and the shape of the male proximal tibiotarsal organ. To describe both species all Neotropical *Sphaeridia* and all described *Denisiella* species were surveyed, presenting notes on both genera, comparative tables, and keys for these taxa.

## ﻿Introduction

Sminthurididae Börner, 1906 is a family of Symphypleona (Collembola) with approximately 155 valid species distributed in one extinct and 12 extant genera (Bellinger et al. 1996–2023). Regarding the living genera, six are monospecific, while *Sphaeridia* Linnaniemi, 1912, *Sminthurides* Börner, 1900 and *Denisiella* Folsom & Mills, 1938 represent the largest lineages in the family, with 69, 60 and 13 valid species, respectively (Bellinger et al. 1996–2023; Ferreira et al. 2021; Medeiros et al. 2022). The family is mainly characterized by the high sexual dimorphism of the antennae, clasper-shaped in males and mostly unmodified in females, the remarkable reduced size of males compared to females, the spherical and short ventral tube sacs, the presence of all abdominal bothriothicha (A–E), and absence of the subanal appendage in females (Richards 1968; Betsch 1980; Bretfeld 1999).

Following Betsch (1980), there are two groups of Sminthurididae: one with a distal tibiotarsal organ on leg III, represented by the current genera *Boernerides* Bretfeld, 1999, *Pedonides* Bretfeld, 2010, *Parasminthurides* Medeiros & Bellini, 2022 (in Medeiros et al. 2022), *Pygicornides* Betsch, 1969, *Sinnamarides* Betsch & Waller, 1991, *Sminthurides*, *Sminthuridia* Massoud & Betsch, 1972, *Stenacidia* Börner, 1906, and *Yosiides* Massoud & Betsch, 1972; and the other without such organ, represented by *Debouttevillea* Murphy, 1965, *Denisiella* and *Sphaeridia. Pseudosminthurides* Sánchez-García & Engel, 2016, the sole extinct genus recorded for the family, has an unknown position between these two groups since such morphology is unclear on its fossil species (Betsch 1980; Sánchez-García and Engel 2016; Medeiros et al. 2022).

While *Denisiella* and *Sphaeridia* may look like closely related genera since they share the absence of the distal tibiotarsal organ on leg III, the absence of subdivisions on the antennal segment IV and of trunk vesicles on males, and the presence of a narrow mucro, they are remarkably different in other features. For instance, the male antennal segments II and III are highly modified in *Denisiella* (vs very slightly modified in *Sphaeridia*), *Denisiella* specimens have a regular unmodified corpus of the ventral tube (vs modified in *Sphaeridia*), and the mucronal chaeta is only present in *Denisiella* (Richards 1968; Betsch 1980; Bretfeld 1999; Palacios-Vargas et al. 2018). Such similarities and discrepancies between these two genera make unclear their relationships with each other and with other genera of Sminthurididae, and the family as well as the Symphypleona as a whole are currently in need of phylogenetic revision (Medeiros et al. 2022; Bellini et al. 2023).

*Denisiella* has eight of its 13 species recorded from the Neotropical Region, while *Sphaeridia* has 46 of its 69 taxa recorded for the same region, which indicates that this is an important domain for the study of these Sminthurididae (Mari Mutt and Bellinger 1990, 1996; Bellinger et al. 1996–2023; Mari Mutt et al. 1997–2021).

Herein we describe a new species of *Denisiella* and another of *Sphaeridia* from Brazil. We also survey the Neotropical taxa of *Sphaeridia* and all species of *Denisiella*, presenting comparative tables, identification keys, and taxonomic notes for each genus.

## ﻿Materials and methods

Specimens of the new species were firstly preserved in 70% ethanol at 6 °C. Afterwards, they were clarified in Nesbitt’s solution, washed in Arlé’s liquid, and mounted on glass slides in Hoyer medium, following the procedures of Arlé and Mendonça (1982) and Jordana et al. (1997) with a few adaptations. Habitus of the two species were photographed in 70% ethanol under a Leica S8APO stereomicroscope attached to a Leica EC4 camera, using LAS v. 4.12 software. Morphological studies and raw drawings were made under a Leica DM750 microscope with a drawing tube attached, and photographs were taken in the same microscope with a Leica MC170 HD camera under LAS v. 4.12 software. Final figures were scanned, and the digital images covered, improved, and organized in plates with CorelDraw 2022 software.

The terminology used in the descriptions follows mainly the studies of Massoud and Betsch (1972) for the male antennal chaetotaxy; Cipola et al. (2014) for the labral chaetotaxy; Betsch and Waller (1994) for the head (including the post-labial region) and anterior large abdomen chaetotaxy; Vargovitsh (2009, 2012, 2013) for the posterior large abdomen chaetotaxy, with adaptations; Betsch (1997) for the small abdomen chaetotaxy; Nayrolles (1988) for the oval and tibiotarsal organs of tibiotarsus III; and Bretfeld (2001) for the dorsal dens chaetotaxy. Drawings and observations were made based on the entire type series.

Abbreviations used in the descriptions and figures:

**Abd** abdominal segment(s);

**Ant** antennal segment(s);

**Th** thoracic segment(s).

In the plates the chaetae present or absent are marked with white arrows; unpaired chaetae on head and trunk are marked with an asterisk. Ant IV subsegments are counted from the base to the apex. Head, trunk (thorax + abdomen), and furcal chaetotaxy are given by half body. Chaetae labels are marked in bold.

The type series of both species are deposited at the Collembola Collection of the Biosciences Center of the Federal University of Rio Grande do Norte (CC/UFRN), Brazil.

## ﻿Results

**Order Symphypleona Börner, 1901**

**Suborder Sminthuridida Bretfeld, 1986 *sensu* Sánchez-García and Engel, 2016**

**Superfamily Sminthuridoidea Börner, 1906 *sensu* Fjellberg, 1989**

**Family Sminthurididae Börner, 1906 *sensu*Medeiros et al., 2022**

### ﻿Genus Sphaeridia

Taxon classificationAnimaliaCollembolaSminthurididae

Linnaniemi, 1912

B7F1B589-9C45-5908-AF88-92FF126E73B9

#### Diagnosis of the genus.

Antennal sexual dimorphism weak, Ant II and III in males only with **b1** and **c3** modified elements, respectively, antennal bothriotricha absent, long sensilla present in both segments. Ant IV undivided in both sexes. Head chaetae usually uniform, sometimes short, and thick. Eyes 5+5 to 8+8, ommatidia C and D small if present. Th III in males without vesicles. Large abdomen bothriotricha **ABC** misaligned. Posterior large abdomen with long chaetae. Ventral tube corpus of males usually modified, with 1+1 extra vesicles (other than the sacs) and/or several complex processes; corpus of females mostly with 1+1 extra vesicles; ventral tube with 0+0 or 1+1 chaetae in both sexes. Tibiotarsi I–II without any clear modifications in both sexes; tibiotarsus III of males usually with modified chaetae **IIpe**, **IIIpi** and **IVpi**; tibiotarsus III of females usually with modified chaetae **IIIpi** and **IVpi**; distal tibiotarsal organ of leg III absent. Ungues I and II usually more slender than the unguis III. Unguiculi I–II with a narrow lamella and a long distal filament, unguiculus III with or without the distal filament, if present, short. Dens dorsally with or without the basal appendage, chaetae **E1** and **J1–3** usually spiniform. Dens ventral chaetotaxy following the formula 2,3,2…1 (rarely 2,2…1 or 2,2,1…1) from the apex to the basis. Mucro narrow, inner edge serrated, outer smooth, without the chaeta (adapted and revised from Krausbauer 1898; Linnaniemi 1912; Salmon 1951; Massoud and Delamare-Deboutteville 1964; Murphy 1965; Richards 1968; Massoud and Betsch 1972; Betsch 1980; Arlé 1984; Mari Mutt 1987; Bretfeld and Gauer 1994; Bretfeld 1995, 1997, 1999, 2002; Bretfeld and Trinklein 2000; Bretfeld and Schulz 2012).

#### Type species.

*Sminthuruspumilis* Krausbauer, 1898.

#### Distribution.

Worldwide (Bretfeld 1999).

#### Remarks.

Here we surveyed and compared the 46 Neotropical species of *Sphaeridia* (Table 1). Many species of the genus are described based mainly in the male ventral tube morphology and leg III modified chaetae, characteristics which are variable and useful for the separation of species (Bretfeld and Gauer 1994; Bretfeld 1997, 1999, 2002; Bretfeld and Trinklein 2000; Bretfeld and Schulz 2012). However, such descriptions lack further data, especially on the antennal, large and small abdomen morphology/chaetotaxy, features which may be useful alone or at least are complementary to distinguish the species.

**Table 1.** Main diagnostic characters of Neotropical *Sphaeridia* species.

Species (known sexes) / characters	Color	Eyes	Frontal head atypical chaetae	Tibiotarsus III - Ipe (♂)	Tibiotarsus III - IIpe (♂)	Tibiotarsus III - IIIpi (♂)	Tibiotarsus III - IVpi (♂)	Tibiotarsus III - IIIpi (♀)	Tibiotarsus III - IVpi (♀)	Dental basal papilla	Dens dorsal chaetae	VT anterior processes (♂)	VT posterior processes (♂)	VT chaetae
*S.aserrata*^3^ (♂,♀)	Dark blue-violet (♂), Pale blue (♀)	8+8	Nc	?	Fk	?	?	Nc	Nc	+	16	?	1+1 vesicles	1+1
*S.aspinosa*^6^ (♂)	Red-violet	?	Nc	?	Sm, Ac, Lg	Tth	Nc	?	?	-?	?	-?	1 small median, 1+1 slender, apical knob processes	1+1
*S.betschi*^2^ (♂,♀)	Dark purple thorax and posterior abdomen, frontal head paler (♂); pale with pigmented Ant IV (♀)	6+6	?	Nc on Pp	Sh, Ls or Nc on Pp	Tth or Fk	Tth or Nc	Sm, Tn	Tth	-	14–17	-	-	?
*S.biclava*^6^ (♂)	Red-violet	?	Nc	?	Sm, Ac, Lg	Tth	Nc or Tth	?	?	-?	?	?	1 thick median, 1+1 thick asymmetrical processes with apexes truncate or knobbed	1+1
*S.biniserrata*^1^,^2,10^ (♂,♀)	Pale purple (♂) Blue-violet, antennal segments dark (♀)	6+6–8+8	Nc	Nc	Fk	Nc	Nc	Tth	Tth	+	16	?	?	?
*S.bivirgata*^7^ (♂)	Large abdomen with two broad blue stripes, Ant III–IV blue	?	Nc	?	Sm, Lg, Ac	Fk	Sm, Th	?	?	+	16	?	1 short blunt median, 1+1 processes with three teeth each, plus several small processes	1+1
*S.boettgeri*^4^ (♂)	Dark blue	?	Th	Nc	Nc	Nc	Nc	?	?	+	10?	1+1 mandible-like processes laterally pointed	1 straight knobbed median process, 1+1 striated blades	1+1
*S.cardosi*^2^ (♂,♀)	Purplish (♂,♀)	6+6	?	Nc	Nc	Nc	Nc	Nc?	Nc?	-?	12?	At least 3+3 processes	1 median, and at least 1 truncated process with a basal appendix	1+1
*S.carioca*^2^ (♂)	Pale with a large black dorsal spot on the large abdomen, antennae dark	6+6	?	Nc	Nc	Fk	Nc	?	?	?	?	At least 3+3 processes	At least 5 processes (1 duck-shaped)	1+1
*S.catapulta*^4^ (♂)	Pale blue	?	Lg	?	Sm, Th	Tth	Tth	?	?	+	16	1+1 thin; 2+2 lateral processes; 1+1 vesicles	1 medial process bifurcated at the apex	1+1
*S.cerastes*^4^ (♂)	Dark blue	?	Th	Nc	Sm, Lg, Th	Nc	Nc	?	?	+	16	1+1 bifurcated processes	1 v-shaped median process, 1+1 knobbed vesicles	1+1
*S.chisacae*^4^ (♂)	Blue, laterally darker	?	?	Nc	Sm, Lg	Nc	Nc	?	?	+	15	1+1 lateral pointed teeth	2 median, 1+1 middle, 1+1 short blunt lateral processes	1+1
*S.clara*^4^ (♂)	Pale	?	Th	?	Nc	Tth	Tth	?	?	+	16	-	1+1 small vesicles	1+1
*S.coronata*^4^ (♂)	Large abdomen with a dark blue horizontal band	?	?	?	Sm, Lg	Tth	Tth	?	?	+	16	1+1 small projections	1+1 small vesicles	1+1
*S.decemdigitata*^8^ (♂)	Blue	?	Nc	Nc	Nc	Tth	Nc	?	?	?	?	?	1 median forked, 5+5 processes (2+2 spines, 1+1 with irregular tips and 2+2 blunt)	-?
*S.delamarei*^5^ (♂)	?	?	Th	?	Bb on Pp	Tth	Tth	?	?	?	?	-	1+1 small vesicles	1+1
*S.denisi*^1,5^ (♂,♀)	Blue-violet, with a pale-yellow background (♂,♀)	8+8	Th	Nc	Bb on Pp	Tth	Tth	Tth	Tth	+	17	-	1+1 small vesicles	1+1
*S.duckei*^7^ (♀)	White with blue lateral and ventral large abdomen, small abdomen dark blue	?	Nc	?	?	?	?	?	?	?	13	?	?	?
*S.fibulifera*^4^ (♂)	Lateral large abdomen dark	?	Sm, Lg, Th	Nc	Sm, Lg	Tth	Tth	?	?	+	16	1+1 small vesicles; 1+1 thick knobs	1+1 long waved processes	1+1
*S.fluminensis*^2^ (♂,♀)	Diffusely lightly pigmented (♂,♀)	6+6	?	Nc	Sm, Cv	Nc	Nc	Fk	Fk	?	?	Asymmetrical bidentate lateral processes	1+1 ring-shaped posterior processes	-
*S.franklinae*^4^ (♂)	Pale blue or brown	?	?	Nc	Sm, Lg	Tth	Tth	?	?	+	16	1+1 lateral small teeth	1 thick medial process with a broad tip, 1+1 lateral curved processes	1+1
*S.gladiolifer*^12^ (♀)	Slightly pigmented dorsally	8+8	Sp	?	?	?	?	Tth	Tth	+	16	?	?	?
*S.heloisae*^2^ (♂,♀)	Dark purple head and body, frontal head Paler (♂); pale with antennal segments purple (♀)	6+6	?	Nc	Ls on Pp	Nc	Tth	Tth	Tth	-?	14–16	1+1 lateral acuminate processes	1 medial, 1+1 hook-like, 1+1acumiante, 1+1 blunt processes	-
*S.irmleri*^4^ (♂)	Deep black	?	?	Nc	Sm, Lg	Tth	Nc	?	?	-	?	1+1 strong borders with 1+1 large doubled teeth	1+1 blades with 3 lobes each, 1+1 lateral processes	-
*S.lobata*^4^ (♂)	Dark blue	?	?	Nc	Sm, Lg	Sm, Sh, Th	Sm, Sh, Th	?	?	+	16	-	1 median process, 2+2 large lobes	1+1
*S.mandibulata*^4^ (♂)	With violet median and horizontal bands, antennae, and legs blue, furca pale	?	Th	Nc	Sm, Lg	Tth	Tth	?	?	+	16	With several symmetrical lobes	1 short median, 1+1 slender lateral, 1+1 blunt tridentate lateral, with a thin protruding membrane hand-glass shaped	1+1
*S.martii*^4^ (♂)	Pale grey	?	Lg	?	Nc	Tth	Tth	?	?	-	21	-	1+1 small vesicles	1+1
*S.multispina*^8^ (♂)	Pale blue	?	Nc	Nc	Nc	Tth	Tth	?	?	?	?	-	-	1+1
*S.neopumilis*^4^ (♂)	Dark blue, paler between eyes and on dorsal and ventral large abdomen	?	Tn	?	Lg, Tn	Sm, Th	Sm, Th	?	?	+	16	-	1+1 small vesicles	1+1?
*S.panguanae*^8^ (♂)	Blue pigment on head and Ant IV	?	Nc	Nc	Sm, Th	Tth	Sm, Th	?	?	?	?	?	1 thick medial, 2+2 acuminate processes	-
*S.paroara*^2^ (♂,♀)	Pale (♂,♀)	5+5–6+6	?	Nc	Sp on Pp	Tth	Tth	Tth	Tth	?	?	1+1 rounded processes	1 median finger-shaped, 1+1 roundish process	1+1
*S.peruensis*^8^ (♂)	Head, body, furca and Ant IV dark blue	?	Nc	Sm, Th	Ls	Fk	Nc	?	?	-	?*	?	1+1 irregular, 1+1 long blunt and several roundish lateral processes	-
*S.pilleata*^4^ (♂)	Large abdomen dorsally blue or with a lateral blue band or entirely blue/brownish/grey or white	?	Lg	?	Nc	Tth	Tth	?	?	+	16	-	1+1 small vesicles	1+1
*S.pippetti*^5,13^ (♂,♀)	Uniform pale, diffused blue	6+6?	Th	Nc	Nc	Tth	Tth	Tth	Tth	+	16	-	1 median process with acutely truncate blunt apex, 1+1 lateral acuminated process	1+1
*S.pumilis*^1,4,12,14,16^ (♂,♀)	Body with or without pale violetish blue mottling (♂,♀)	8+8	Sp(+/-)	Nc	Sm, Th, Lg	Nc	Nc	Tth	Tth	+	16	-	1+1 small vesicles	1+1
*S.robusta*^4^ (♂)	Dark blue	?	Th, Lg	?	Sm, Th on Pp	Tth	Tth	?	?	+	16	-	1+1 small vesicles	1+1
*S.schalleri*^1,5^ (♂,♀)	Pale violet (♂); violet (♀)	8+8	Sh, Th	Sm, Th, Lg	Sm, Th, Lg	Sm, Th	Sm, Th	Tth	Tth	+	17	?	2 median posterior processes, the anterior with 2 teeth, 1+1 lateral processes	1+1
*S.serrata*^9,11,15^ (♀)	Brown or reddish-brown	8+8?	?	Nc?	Nc?	Nc?	Nc?	Tth	Tth	?	10?	?	5 distal processes?	?
*S.squamifera*^4^ (♂)	Grey-blue	?	?	?	Th, Bb on Pp	Tth	Tth	?	?	+/-	16	1+1 small processes	1+1 small vesicles	1+1
*S.spira*^4^ (♂)	Pale with a lateral violet band or spots	?	Th	Lg, Da	Lg, Da	Lg, Tth	Th, Sm	?	?	-	16	Asymmetrical bent lobe bent from the right to the left	1 small median process, asymmetrical membrane, twisted lobe and a basal spine	-
*S.sturmi*^4^ (♂)	With a horizontal band and ventral side blue	?	?	Lg, Da	Lg, Da	Th, Tth	Th, Sm	?	?	-	16	?	3–5 finger-like medial, 1+1 medial asymmetrical, 1+1 lateral thin processes	1+1
*S.torifera*^8^ (♂)	Head and body with small blue spots	?	Nc	Sm, Th	Ls or vesicle	Tth	Tth	?	?	?	?	?	1 blunt median, 1+1 irregular distal processes	1+1
*S.tropica*^8^ (♂)	Head pale blue, body blue, Ant IV dark blue	?	Nc	Nc on Pp	Sm, Cv on Pp	Sm, Th	Tth	?	?	?	?	?	1 median bladder-like, 1+1 finger-like spines processes, 1+1 irregular lobes	1+1
*S.tschirnhausi*^8^ (♂)	Body and extremities dark violet-brown	?	Nc	Sm on Pp	Bb	Tth	Tth	?	?	?	?	1+1 roundish notched lobes	-	1+1
*S.vampyra*^8^ (♂)	Entirely blue or with pale dorsal sides	?	Nc	Nc	Sm, Tn, Lg	Nc	Nc	?	?	?	?	?	1 thick and blunt medial, 2+2 pointed processes	-
*S.winteri*^1,5^ (♂,♀)	Blue-violet, with a pale-yellow background (♂,♀)	8+8	Sh, Th	Nc	Sm, Lg, Tn on Pp	Sm, Th, Lg, Bt	Sm, Sh, Th	Tth	Tth	+	17	1+1 large lateral teethed, 1+1 small processes	2 median processes, the anterior with a posterior tooth, 1+1 small lateral processes	1+1
*S.piauiensis***sp. nov.** (♂,♀)	Pale purple body, dark purple antennae (♂)	8+8	Nc	Nc	Ls on Pp	Nc	Nc	Tth	Tth	+	15	At least 3+3 anterior projections	At least 8+8 posterior projections, two of them striated	1+1

**Figure 1.**
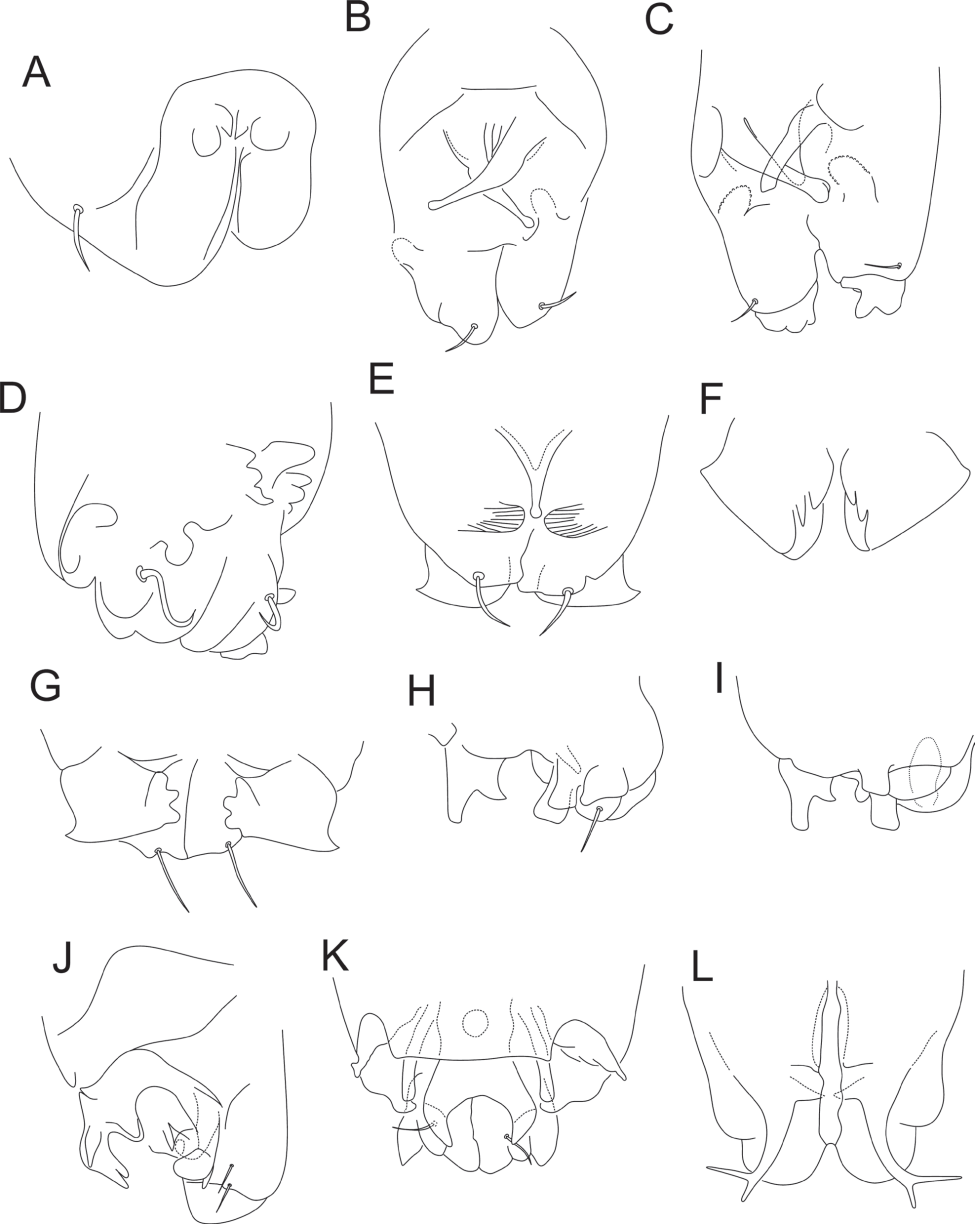
Morphology of the ventral tube of males of *Sphaeridia* spp. **A** apex in *S.aserrata* Mari-Mutt, 1987 **B** posterior side in *S.aspinosa* Bretfeld & Trinklein, 2000 **C** posterior side in *S.biclava* Bretfeld & Trinklein, 2000 **D** posterior side in *S.bivirgata* Bretfeld, 2002 **E** posterior side in *S.boettgeri* Bretfeld & Gauer, 1994 **F** Detailed view of the anterior mandible-like processes of *S.boettgeri***G** anterior side (apex) in *S.boettgeri***H** apex in *S.cardosi* Arlé, 1984 **I** apex in *S.cardosi* (specimens from a different locality) **J** apex in *S.carioca* Arlé, 1984 **K** posterior side in *S.catapulta***L** anterior side in *S.cerastes* Bretfeld & Gauer, 1994. Figures adapted from species’ original descriptions.

**Figure 2.**
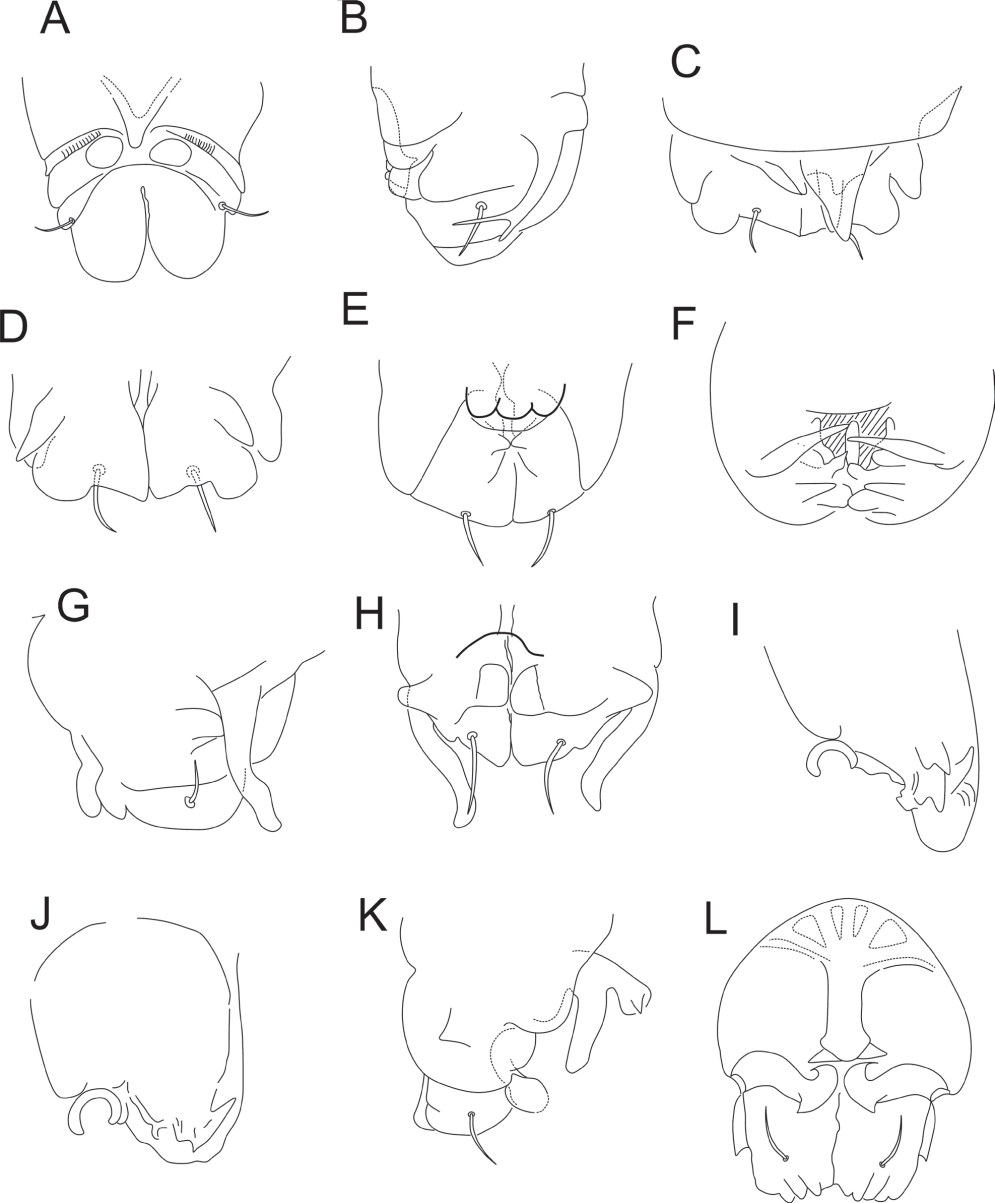
Morphology of the ventral tube of males of *Sphaeridia* spp. (cont.) **A** posterior side in *S.cerastes***B** apex in *S.cerastes***C** anterior side in *S.chisacae* Bretfeld & Gauer, 1994 **D** posterior side in *S.chisacae***E** posterior side in *S.coronata* Bretfeld & Gauer, 1994 **F** posterior side in *S.decemdigitata* Bretfeld & Schulz, 2012 **G** apex in *S.fibulifera* Bretfeld & Gauer, 1994 **H** anterior side in *S.fibulifera***I** apex in *S.fluminensis* Arlé, 1984 **J** apex in *S.fluminensis* (another view) **K** apex in *S.franklinae* Bretfeld & Gauer, 1994 **L** posterior side in *S.franklinae*. Figures adapted from species’ original descriptions.

**Figure 3.**
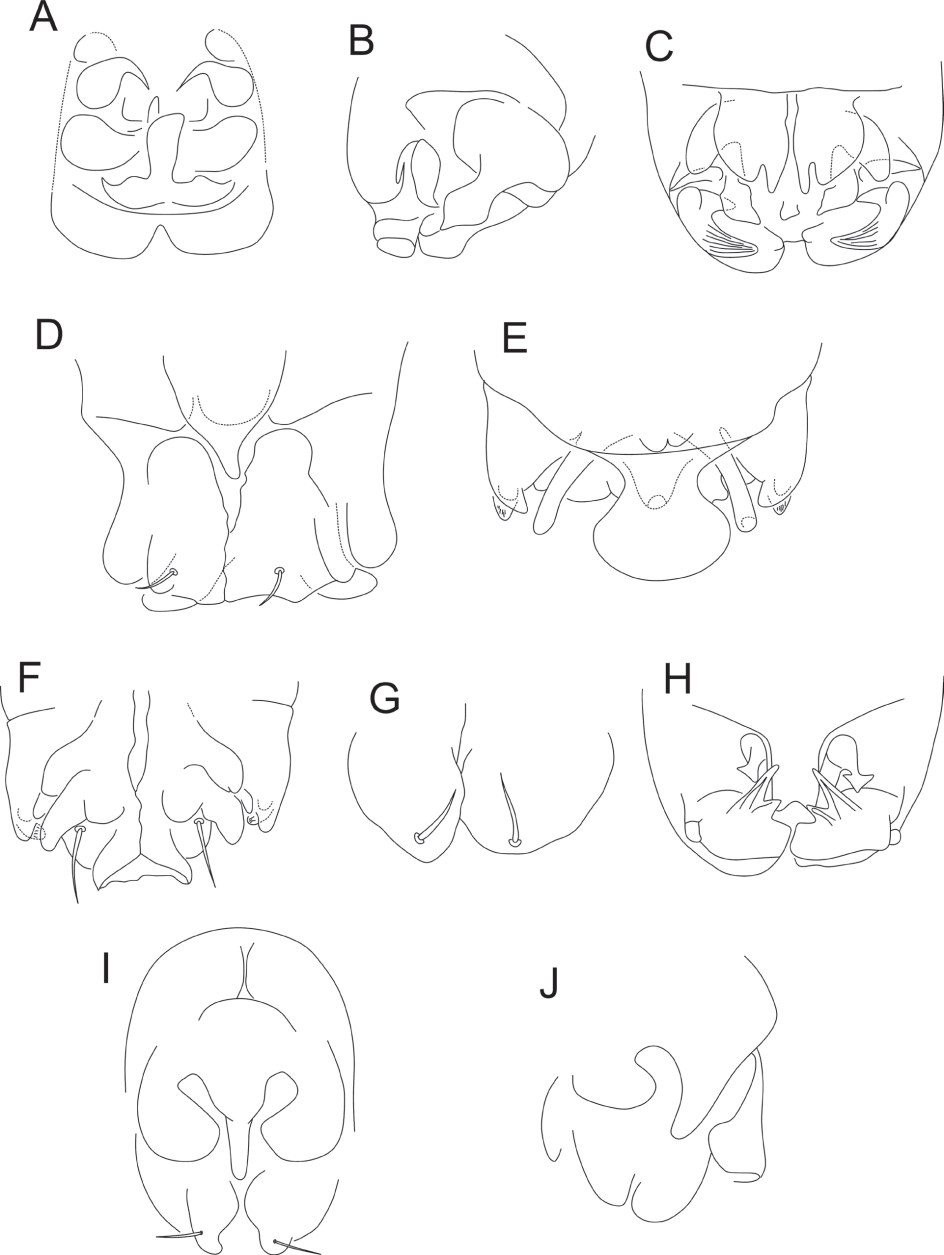
Morphology of the ventral tube of males of *Sphaeridia* spp. (cont.) **A** apex in *S.heloisae* Arlé, 1984 **B** apex in *S.heloisae* (another specimen) **C** posterior side in *S.irmleri* Bretfeld & Gauer, 1994 **D** posterior side in *S.lobata* Bretfeld & Gauer, 1994 **E** posterior side in *S.mandibulata* Bretfeld & Gauer, 1994 **F** anterior side in *S.mandibulata***G** posterior side in *S.multispina* Bretfeld & Schulz, 2012 **H** posterior side in *S.panguanae* Bretfeld & Schulz, 2012 **I** posterior side in *S.paroara* Arlé, 1984 **J** apex in *S.paroara*. Figures adapted from species’ original descriptions.

**Figure 4.**
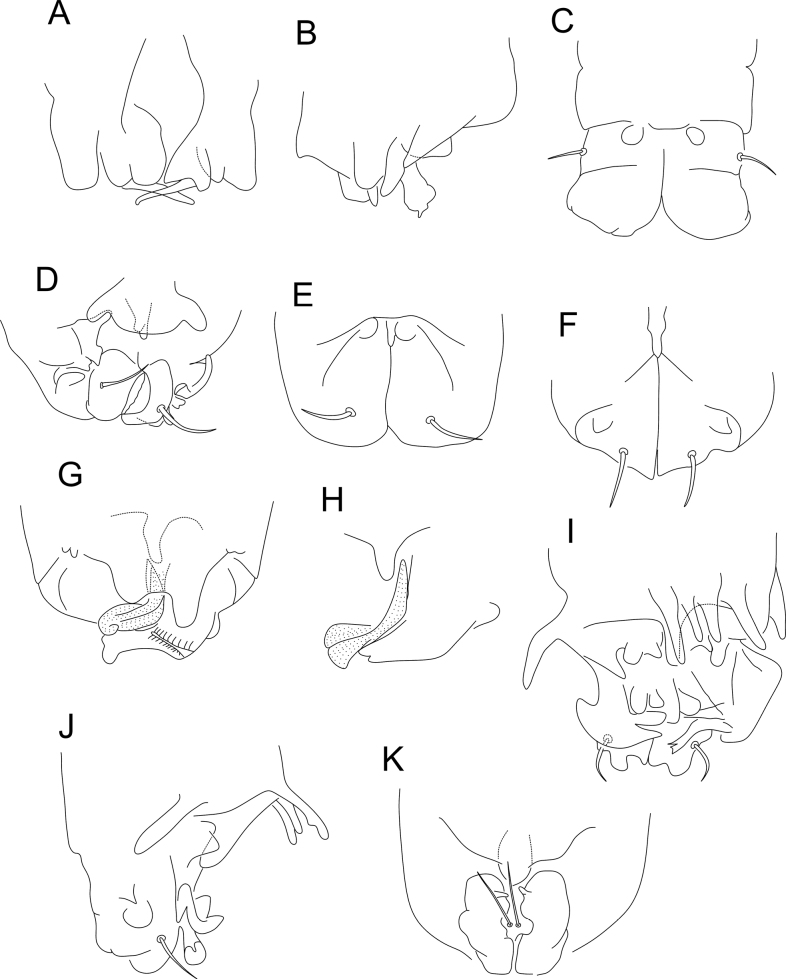
Morphology of the ventral tube of males of *Sphaeridia* spp. (cont.) **A** posterior side in *S.peruensis* Bretfeld & Schulz, 2012 **B** apex in *S.peruensis***C** posterior side in *S.pilleata* Bretfeld & Gauer, 1994 **D** posterior side in *S.schalleri* Massoud & Delamare-Deboutteville, 1964 **E** posterior side in *S.squamifera* Bretfeld & Gauer, 1994 **F** anterior side in *S.squamifera***G** apex in *S.spira* Bretfeld & Gauer, 1994 **H** apex in *S.spira*, detailed view of the two asymmetrical lobes **I** posterior side in *S.sturmi* Bretfeld & Gauer, 1994 **J** apex in *S.sturmi***K** posterior side in *S.torifera* Bretfeld & Schulz, 2012. Figures adapted from species’ original descriptions, with the exception of *S.schalleri* which was adapted from Bretfeld (1997).

**Figure 5.**
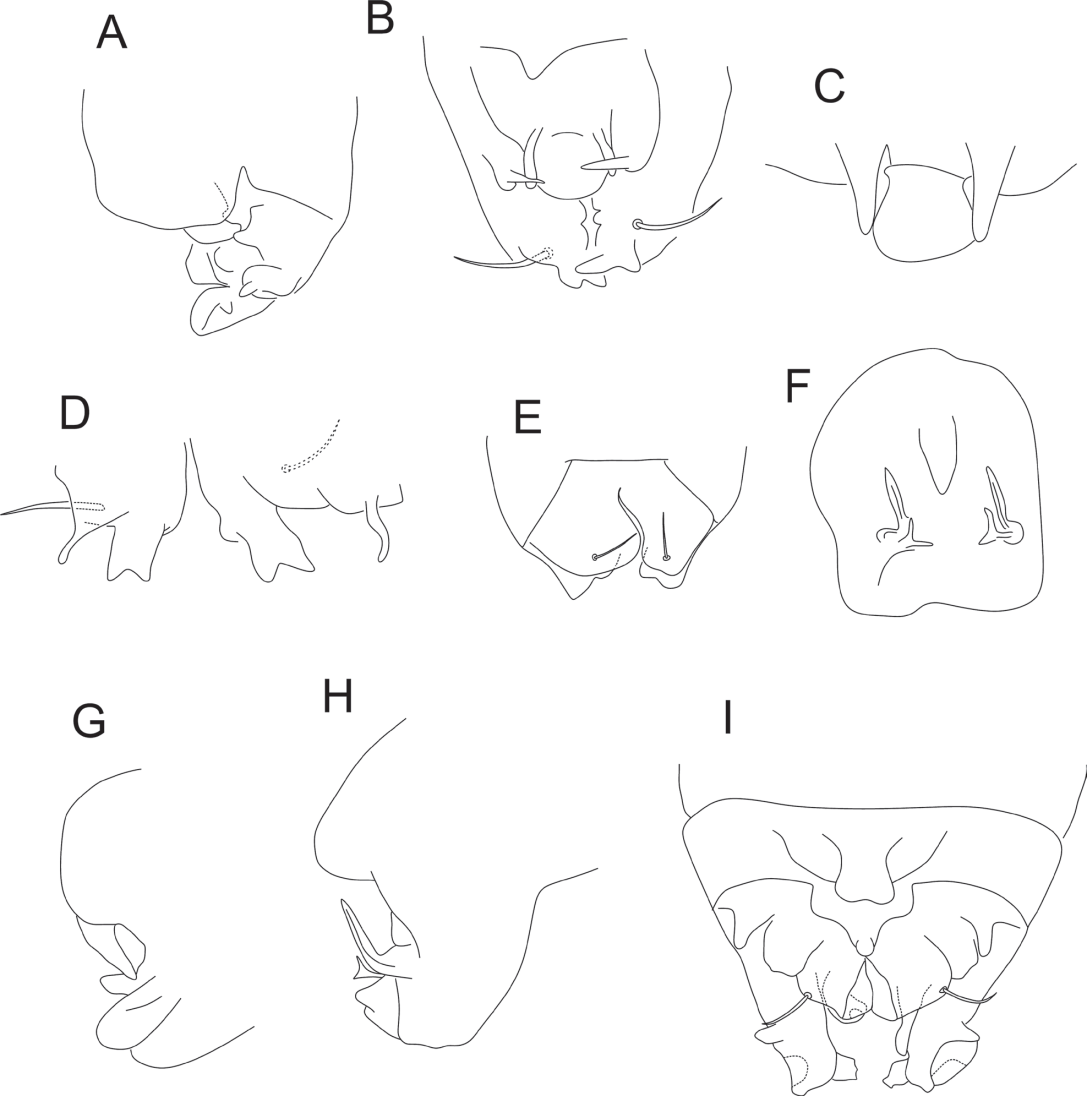
Morphology of the ventral tube of males of *Sphaeridia* spp. (cont.) **A** apex in *S.torifera***B** posterior side in *S.tropica* Bretfeld & Schulz, 2012 **C** detail of the apex of the posterior side in *S.tropica***D** detail of the apical lobes in *S.tropica***E** posterior side in *S.tschirnhausi* Bretfeld & Schulz, 2012 **F** posterior side in *S.vampyra* Bretfeld & Schulz, 2012 **G** lateral side in *S.vampyra*, detail of the posterior median plane **H** lateral side in *S.vampyra*, detail of the posterior lateral plane **I** posterior side in *S.winteri* Massoud & Delamare-Deboutteville, 1964. Figures adapted from species’ original descriptions, with the exception of *S.winteri* which was adapted from Bretfeld (1997).

Data based in: main bibliography: ^1^Massoud and Delamare-Deboutteville (1964); ^2^Arlé (1984); ^3^Mari Mutt (1987); ^4^Bretfeld and Gauer (1994); ^5^Bretfeld (1997); ^6^Bretfeld and Trinklein (2000); ^7^Bretfeld (2002); ^8^Bretfeld and Schulz (2012); supplementary bibliography: ^9^Folsom and Mills (1938); ^10^Salmon (1951); ^11^Stach (1956); ^12^Delamare-Deboutteville and Massoud (1964); ^13^Murphy (1966); ^14^Bretfeld (1995); ^15^Christiansen and Bellinger (1998); ^16^Bretfeld (1999). Legends: + = present; - = absent; ? = unclear/unknown; Ac = acuminated; Bb = bipartite blade; Bl = blunt chaeta/lobes; Cv = curved; Da = Dagger-like; Fk = forked; Ht = Hooked tip; Lg = Long; Ls = Leaf-shaped; Nc = normal chaeta; Pp = papilla; Sm = Smooth; Sp = spiniform; Sh = Short; Th = thick; Tn = thin; Tth = Tooth; * = with 2+2 furcal hooks. A detailed view of the male ventral tube morphology is presented in Figs 1–5 and 11A.

According to Bretfeld and Gauer (1994), *Sphaeridia* holds four groups of species separated by the male ventral tube morphology (Table 1, Figs 1–5). The *pumilis* group is characterized by species usually with only 1+1 small posterior vesicles or rarely lacking such structures, without further modifications on the ventral tube (Figs 1A, 2E, 3G, 4C, 4E–F, 5E); the *brevipila* group is characterized by taxa with a posterior median process (usually with extra processes as well) on the ventral tube (Figs 1B–L, 2C, D, 2F, 2I–L, 3A, B, 3D–F, 3H–J, 4D, 4K, 5A–D, 5F–I); the *irmleri* group is represented by species with posterior and lateral complex symmetrical processes on the ventral tube (Figs 2G, H, 3C, 4A, B); and the *spira* group is characterized by taxa with complicated asymmetrical structures on the ventral tube (Fig. 4G–J), combined with extra modified chaetae on male tibiotarsus III.

In our survey of the Neotropical *Sphaeridia* we noticed that at least eight species do not have enough characters to clearly differ them from other congeners or to keep them in the genus, and so we are considering them as *species inquerendae*. These taxa are *S.aspinosa* Bretfeld & Trinklein, 2000, *S.biniserrata* (Salmon, 1951) *sensu* Massoud & Delamare-Deboutteville (1964), *S.delamarei* Bretfeld, 1997, *S.duckei* Bretfeld, 2002, *S.gladiolifer* Delamare-Deboutteville & Massoud, 1964, *S.martii* Bretfeld & Gauer, 1994, *S.multispina* Bretfeld & Schulz, 2012 and *S.serrata* (Folsom & Mills, 1938). *Sphaeridiaaspinosa* and *S.multispina* males do not present the typical clasper organ with modified Ant II and III chaetae seen in all other Sminthurididae. Bretfeld and Trinklein (2000: 190) and Bretfeld and Schulz (2012: 515) suggested these specimens could be anomalous, but they had few specimens of both species to confirm such condition as abnormal. Also Bretfeld and Trinklein (2000: 190) suggested the absence of the clasper could imply that *S.aspinosa*, and consequently *S.multispina*, may belong to an undescribed different basal genus of the family, which makes these species not comparable with the other taxa of *Sphaeridia* and their descriptions in need for a revision. *Sphaeridiaduckei* was described based on a single female with different features from those seen in all other Sminthurididae, like capitate tenent hairs in all pairs of legs and trunk bothriotricha ABC aligned, which may indicate this specimen may be a subadult of another family, like Bourletiellidae. It is worth noting that Bretfeld (1999:52) suggested that the records of the type species of *Sphaeridia* must be confirmed with males, because the females do not show differences in many species, supporting our view of *S.duckei*. On the other hand, *S.biniserrata*, *S.delamarei*, *S.gladiolifer* and *S.serrata* likely belong to *Sphaeridia*, but their descriptions lack sufficient information about the antennae, legs and/or ventral tube to clearly separated them from other taxa, which makes comparisons with other species arbitrary (see Table 1). Finally, *S.martii* does not fit in *Sphaeridia* since the morphology of its male antennae is more complex compared to other taxa, presenting modified chaetae **b1–b**3 on Ant II and **c1–c3** on Ant III. Even with this observation, its placement among the Sminthurididae is still unclear. So we believe all these species need redescriptions and a revision of their placement within the family.

### ﻿ Sphaeridia piauiensis

Taxon classificationAnimaliaCollembolaSminthurididae

Medeiros & Bellini sp. nov.

C872CB77-A996-5C57-B90F-34A0C16B8F00

https://zoobank.org/3A3FB9DF-117B-4E36-B195-C097D270C357

**Figure 6.**
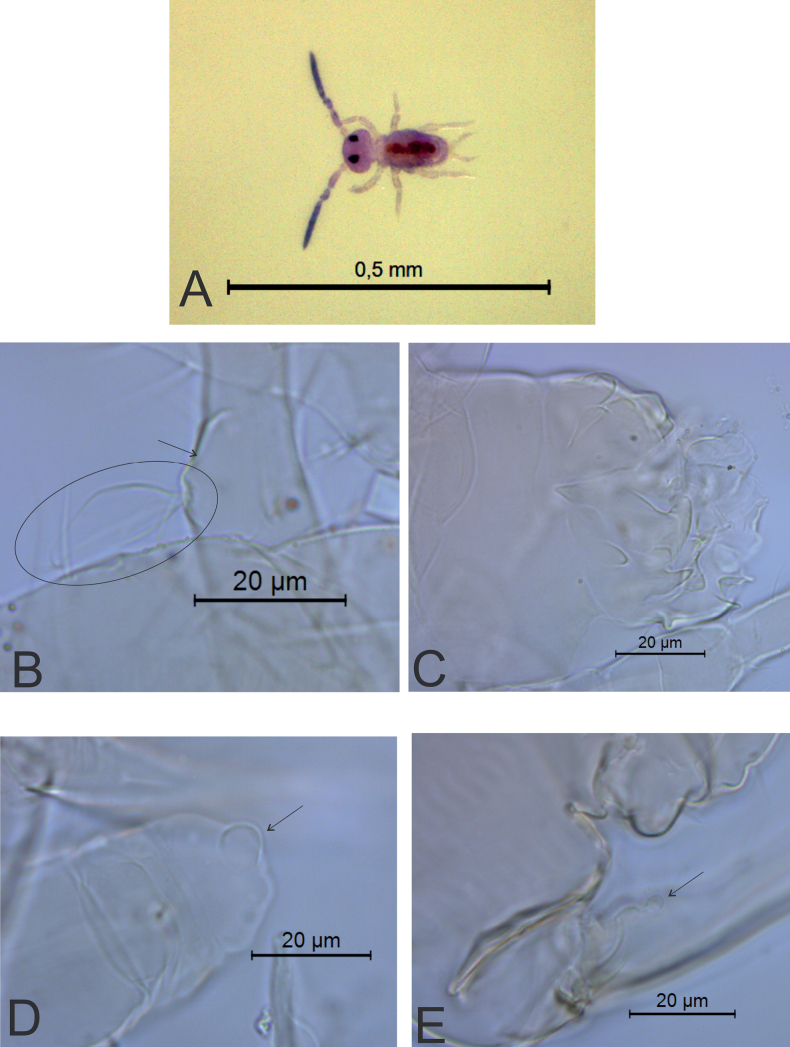
*Sphaeridiapiauiensis* sp. nov. **A** habitus of a male in ethanol (dorsal view) **B** leaf-shaped chaeta on dorsal tibiotarsus III of a male **C** male ventral tube (apex) **D** female ventral tube (apex), arrow points to a vesicle **E** female dens, arrow points to the basal appendage.

**Figure 7.**
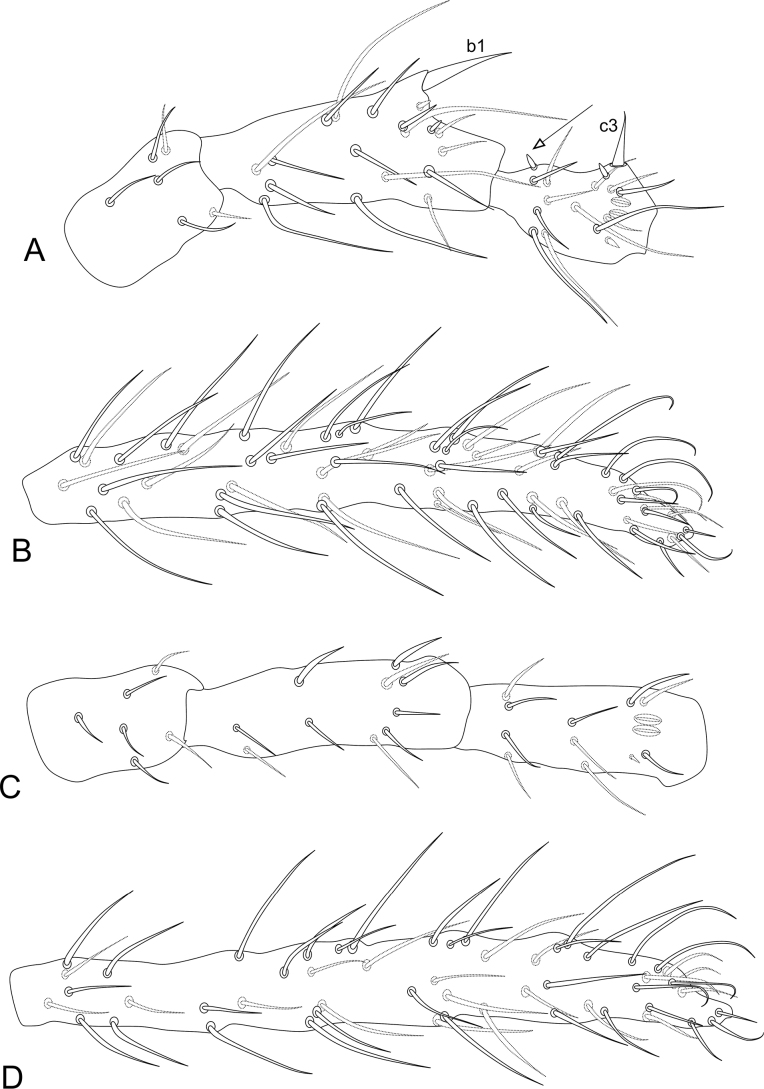
*Sphaeridiapiauiensis* sp. nov. antenna (dorsal view) **A, B** male antenna **A**Ant I–III **B**Ant IV **C, D** female antenna **C**Ant I–III **D**Ant IV.

**Figure 8.**
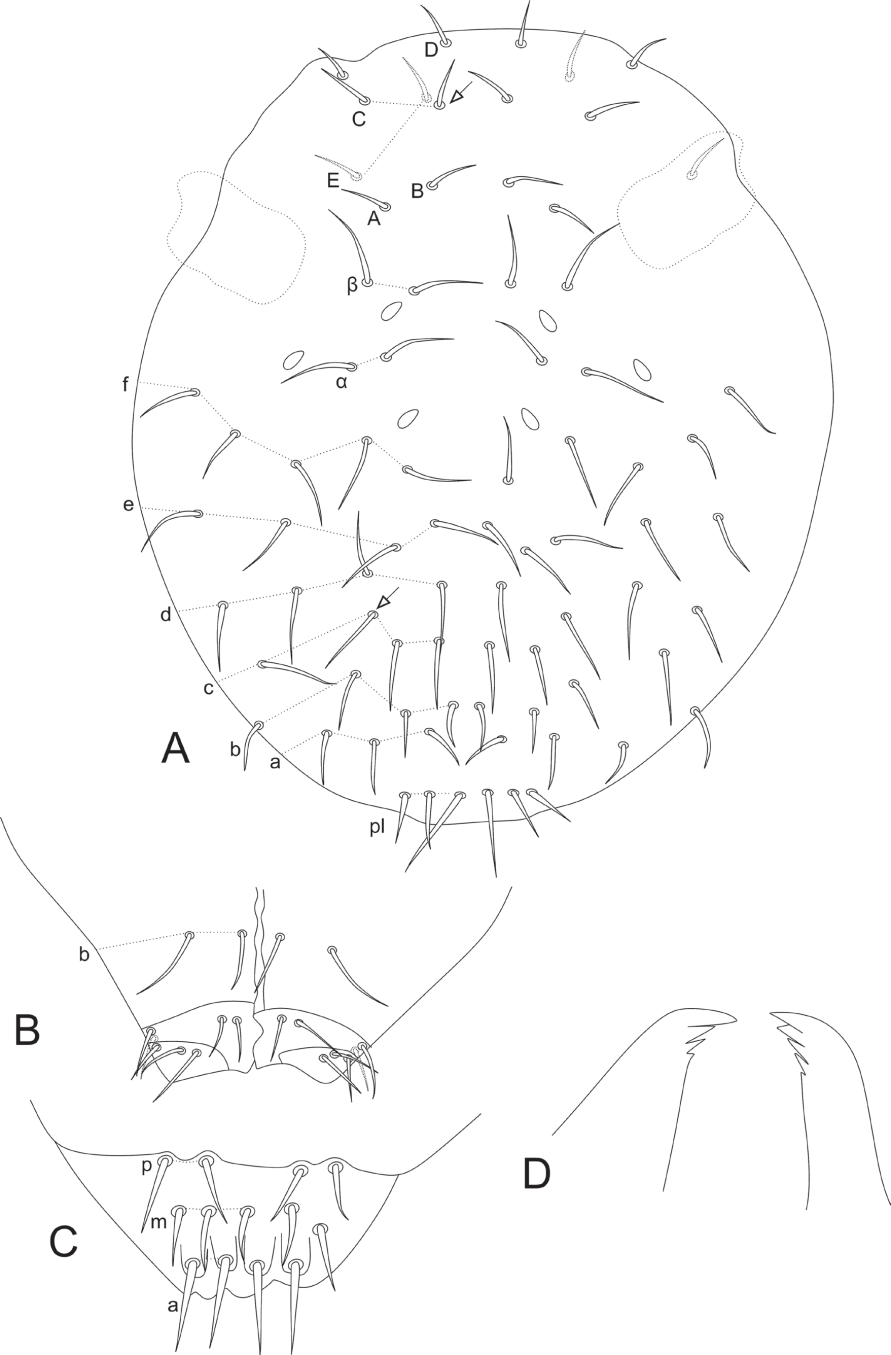
*Sphaeridiapiauiensis* sp. nov. head **A** male anterior head chaetotaxy (eyes omitted), arrow points to a chaeta which can be present or absent **B** male labial and post-labial (ventral) chaetotaxy **C** male labral chaetotaxy **D** male mandibles apexes (incisive teeth).

**Figure 9.**
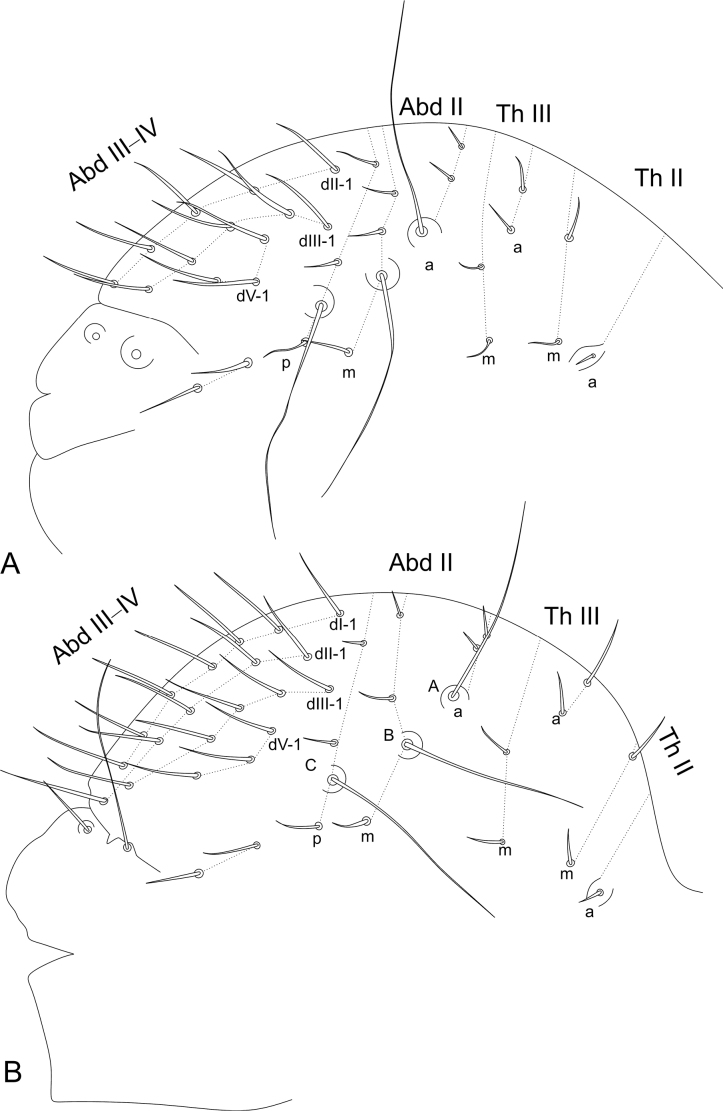
*Sphaeridiapiauiensis* sp. nov. large abdomen **A** male **B** female.

**Figure 10.**
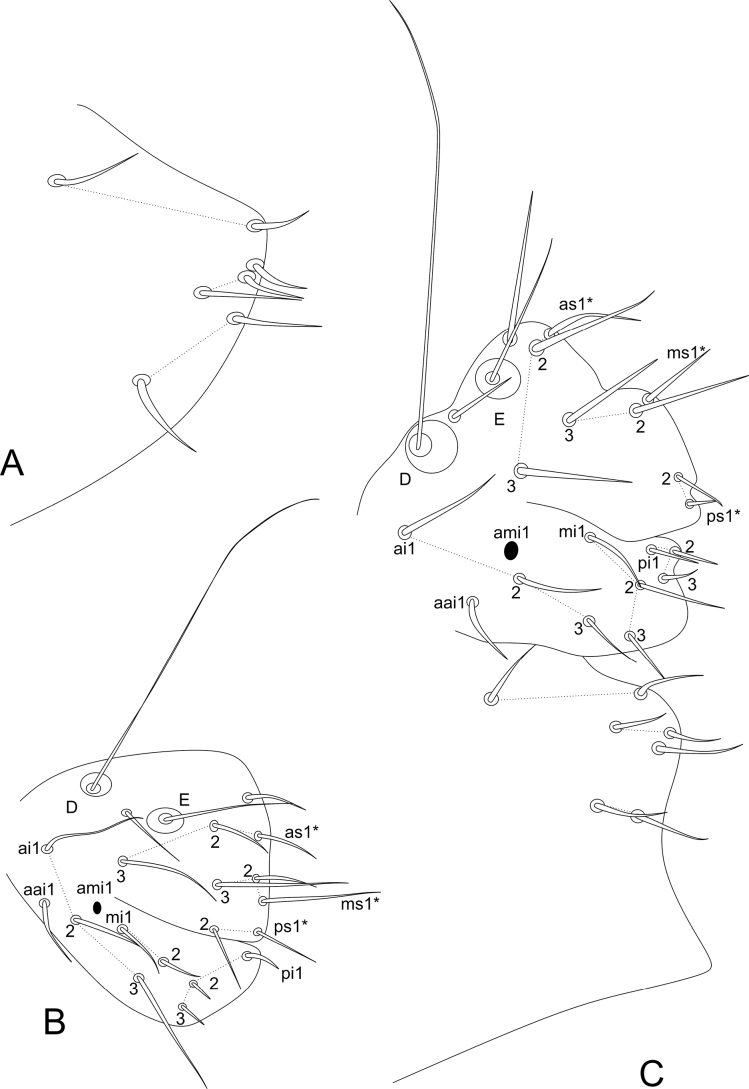
*Sphaeridiapiauiensis* sp. nov. parafurcal area and small abdomen **A** male parafurcal area **B** male small abdomen **C** female parafurcal area and small abdomen.

**Figure 11.**
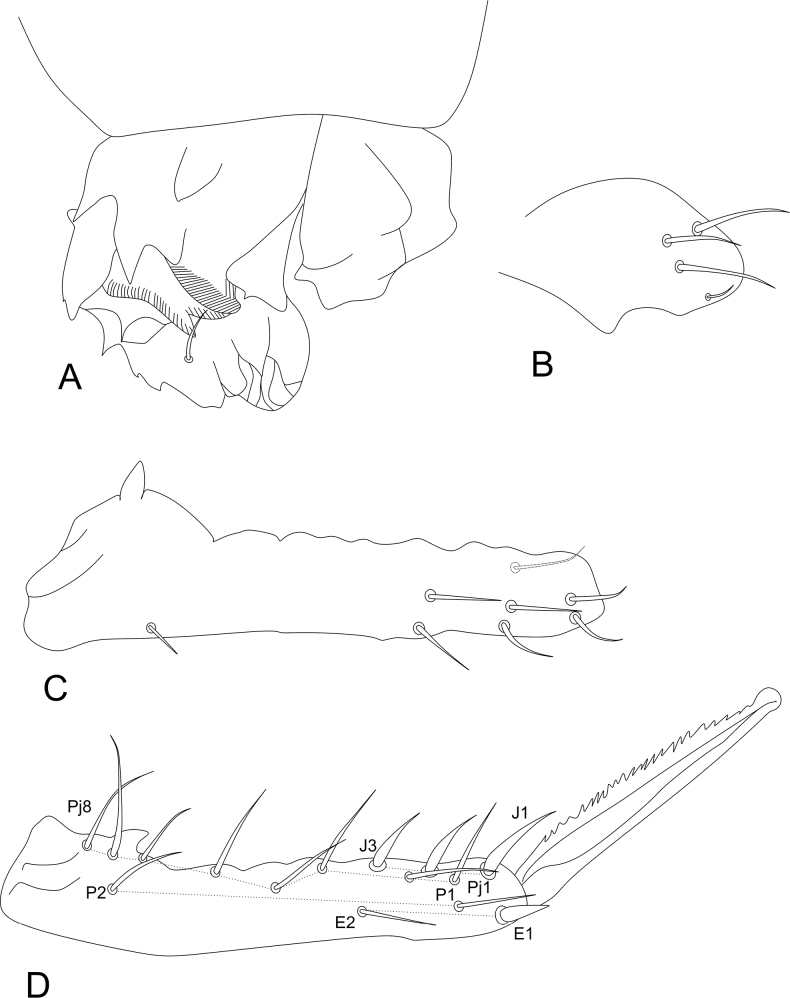
*Sphaeridiapiauiensis* sp. nov. male abdominal appendages **A** ventral tube (lateral view) **B** manubrial chaetotaxy (lateral view) **C** ventral dens chaetotaxy **D** dorsal dens chaetotaxy and mucro.

**Figure 12.**
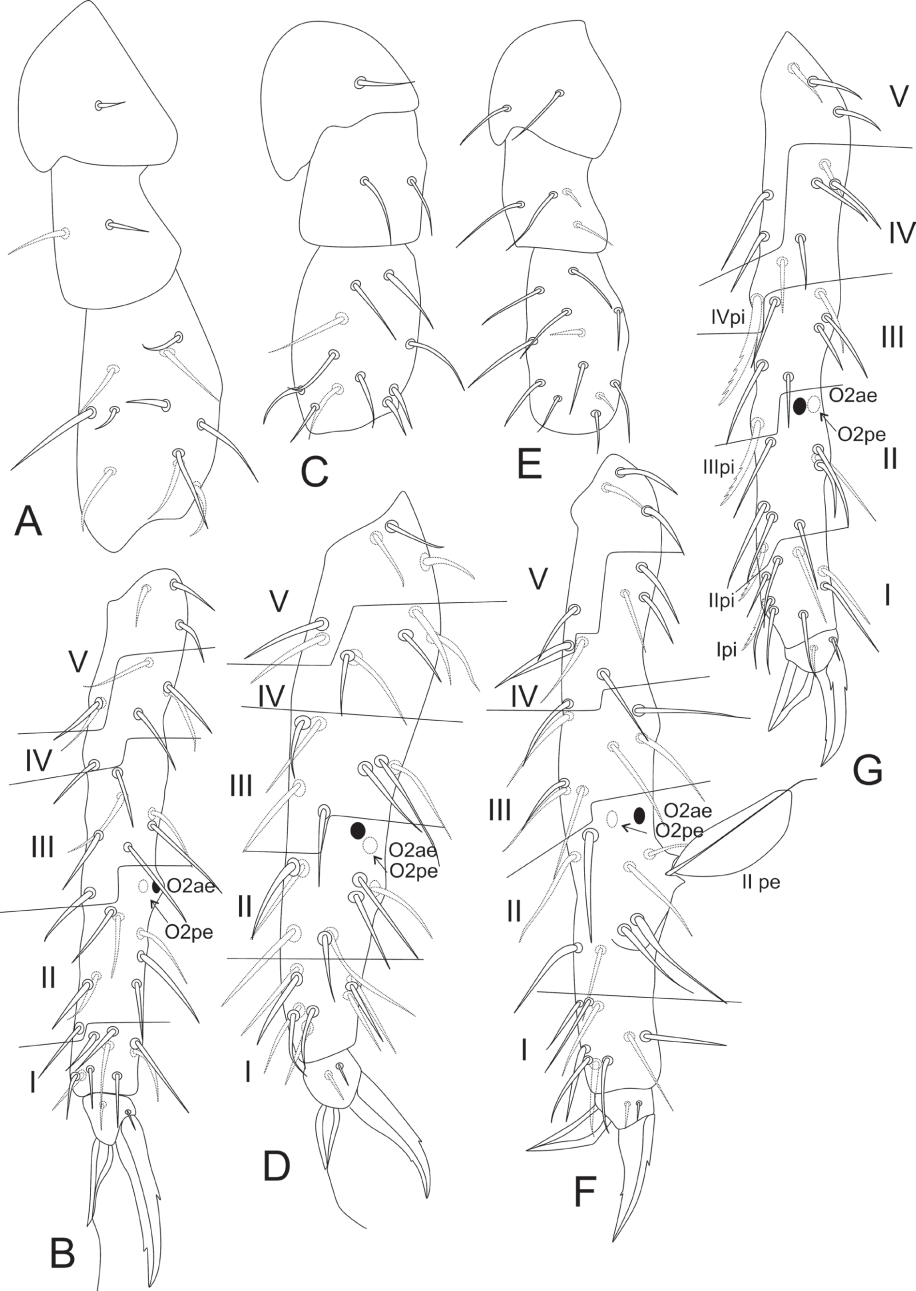
*Sphaeridiapiauiensis* sp. nov. male legs **A** coxa, femur, and trochanter of leg I **B** tibiotarsus and empodial complex of leg I **C** coxa, femur, and trochanter of leg II **D** tibiotarsus and empodial complex of leg II **E** coxa, femur and trochanter of leg III **F** tibiotarsus and empodial complex of leg III **G** tibiotarsus and empodial complex of leg III of the female.

Figs 6 , 7 , 8 , 9 , 10 , 11 , 12 , Table 1

#### Type material.

***Holotype*** male on slide, Brazil, Piauí state, Piracuruca municipality, Sete Cidades National Park, ‘Primeira Cidade’ (4°05'42.53"S, 41°40'50.7"W), 168 m, in sandy soil, ecotonal zone between Caatinga and Cerrado biomes, 14/V/2021, A.M.N. Silva col., pitfall traps. ***Paratypes*** on slides: one male, one female, and one juvenile, with the same data of the holotype.

#### Diagnosis.

Male specimens with a pale purple body, antennae dark purple (Fig. 6A). Males Ant II with two spiniform microsensilla plus six long chaetae other than **b1**, Ant III with two or three spiniform microsensilla plus four long chaetae other than **c3.** Head without any medial chaeta, with 1+1 zones without cuticular granulation between the antennae. Medial prelabral chaetae longer than others in both sexes. Femur I and III with two and one curved chaetae, respectively. Tibiotarsus III of males with a IIpe chaeta leaf-shaped inserted in a large papilla (Fig. 6B), tibiotarsus III of females with **Ipi**, **IIpi**, **IIIpi**, and **IVpi** chaetae serrated. Ungues I–III without tunica or pseudonychia. Dorsal anal valve with three unpaired chaetae (**as1**, **ms1**, and **ps1**). Parafurcal area with seven chaetae. Tenaculum with 1+1 chaetae. Ventral tube in males highly modified with several apical processes, two of them in the posterior region lamellated (striated), with 1+1 chaetae (Fig. 6C), female ventral tube with 1+1 apical vesicles plus 1+1 chaetae (Fig. 6D). Dorsal dens with a basal appendage (Fig. 6E), **J1–3** and **E1** chaetae spiniform, ventral formula as 2,3,2...1 from the apex to the basis.

#### Description.

***Body*** (head + trunk) length of the type series ranging between 228 and 571 µm, holotype with 269 µm, male average size = 248 µm, female average size = 280 µm, entire type series average size = 259 µm. Male specimens with a pale purple body, antennae dark purple (Fig. 6A). Female color pattern unknown (studied specimen already on a glass slide).

***Head*** (Figs 7, 8). Antennae length 202 µm in the holotype. Holotype antennal segment ratio I:II:III:IV as 1:2.3:1.7:5.9. Males’ antenna: Ant I with six chaetae (Fig. 7A). Ant II with element **b1** as a thick spine in a large papilla, plus 16 regular chaetae, six of them longer than others (three of them on basal half and three of them on distal half), plus two spiniform microsensilla (Fig. 7A). Ant III with element **c3** present as a thick spine in a papilla, plus 12 regular chaetae, four of them longer than others (two on basal half and two on distal half), plus two or three spiniform microsensilla; apical organ sensory rods in two independent shallow invaginations (Fig. 7A). Ant IV longer than Ant III, undivided, with ~ 61 chaetae, ~ 8 of them as a curved subapical sensilla (Fig. 7B). Females’ antenna: Ant I with six chaetae (Fig. 7C). Ant II with 10 chaetae (Fig. 7C). Ant III with 10 chaetae, plus one apical spiniform microsensilla; apical organ sensory rods in two independent shallow invaginations (Fig. 7C). Ant IV longer than Ant III and undivided, with ~ 54 chaetae, ~ 7 of them as curved subapical sensilla (Fig. 7D). Head capsule (both sexes): Eyes 8+8 plus one interocular chaeta, head capsule normal (not elongated) (Fig. 8A). Clypeal area **a–f** lines with 3/6/3–4/4/4/5 dorsal + ventral chaetae, respectively (Fig. 8A, B). Interantennal area **α** and **β** lines with 2/2 chaetae, respectively; frontal area **A–E** lines with 1/1/1–2/1/2 chaetae, respectively; 1+1 interocular chaetae present; 1+1 zones without cuticular granulation present between the antennae (Fig. 8A). Labial basomedian and basolateral fields with three chaetae each (Fig. 8C). Six prelabral chaetae present, medial chaetae longer and thicker than the others (Fig. 8C); labral **p**, **m**, and **a** lines with 4, 5, 4 chaetae, respectively; each chaeta of **a** line in an individual papilla (Fig. 8D). Mandibles normal (not elongated), with 4+5 incisive apical teeth (Fig. 8E). Maxillae and labial palp papillae unclear. Anterior head, labrum, labium, and visible mouthparts without any clear sexual dimorphism.

***Trunk*** (Figs 9, 10). Large abdomen: thorax continuous with the abdomen, without clear segmentations in both sexes. Males: Th II with one **a** chaeta inside a cavity and two **m** chaetae; Th III with two **a** and two **m** chaetae; Abd I without chaetae, Abd II with bothriotricha **A**, **B**, and **C** slightly misaligned, with two **a**, three **m**, and three **p** regular chaetae near the bothriotricha; Abd III–IV with three main lines of chaetae above the bothriotrichum **C**: **dII-1** with five, **dIII-1** with five, **dV-1** with three, plus two lateral chaetae under the bothriotrichum **C** (Fig. 9A). Parafurcal area with four rows of chaetae with two, two, one, and two (total of seven) chaetae, neosminthuroid chaetae absent (Fig. 10A). Females: Th II with one **a** chaeta inside a cavity and two **m** chaetae; Th III with two **a** and two **m** chaetae; Abd I without chaetae, Abd II with bothriotricha **A**, **B**, and **C** slightly misaligned, with two **a**, three **m**, and three **p** regular chaetae near the bothriotricha; Abd III–IV with four main lines of chaetae above the bothriotrichum **C**: **dI-1** with seven, **dII-1** with five, **dIII–1** with five, **dV–1** with three, plus two lateral chaetae under the bothriotrichum C (Fig. 9B). Parafurcal area with four rows of chaetae with two, two, one, and two (total of seven) chaetae, neosminthuroid chaetae absent (Fig. 10C). Small abdomen: including Abd V–VI in both sexes. Males: Abd V with bothriotricha **D** and **E** present, plus two regular chaetae. Dorsal anal valve with **as1–3**, **ms1–3**, and **ps1–2** chaetae, **as1**, **ms1**, and **ps1** unpaired; each ventral anal valve with **aai1**, **ai1–3**, **ami1** (as an oval organ lacking any inner sensillum), **mi1–2**, **mpi1**, and **pi1–3** chaetae (Fig. 10B). Females: Abd V with bothriotricha **D** and **E** present, plus two regular chaetae. Dorsal anal valve with **as1–3**, **ms1–3**, and **ps1–2** chaetae, **as1**, **ms1**, and **ps1** unpaired; each ventral anal valve with **aai1**, **ai1–3**, **ami1** (as an oval organ lacking any inner sensillum), **mi1–3**, **mpi1**, and **pi1–3** chaetae (Fig. 10C). Genital plate chaetotaxy unclear in both sexes.

***Abdominal appendages*** (Fig. 11). Ventral tube in males symmetrical, with at least three anterior projections and at least eight posterior projections, two of them lamellated (striated), with 1+1 distal chaetae on lateral flaps (Fig. 11A), females with 1+1 vesicles and 1+1 distal chaetae (Fig. 6D). Tenaculum ramus with three teeth, each plus an apically rounded basal appendix, corpus with 1+1 chaetae. Manubrium with four dorsal chaetae (Fig. 11B); dens with a basal appendage and 15 dorsal (posterior) chaetae, lines **E/P/PJ/J** with 2/2/8/3 chaetae, respectively; **J** line and **E1** chaeta as thick acuminated spiniform chaetae (Fig. 11D); dens ventrally (anteriorly) with eight chaetae, following the formula from the apex to the basis: 2,3,2...1 (Fig. 11C). Mucro narrow with a broad apex, with an external lamella smooth and an internal lamella serrated with ~ 22 teeth; mucronal chaeta absent (Fig. 11D). Furca without any clear sexual dimorphism. Manubrium:dens:mucro ratio of the holotype = 3.3:6.4:1.

***Legs*** (Fig. 12). Leg I: coxa with one chaeta; trochanter with two chaetae; femur with 11 chaetae, two of them as curved chaetae; tibiotarsus with 34 chaetae and two oval organs (**O2pe**, **O2ae**), whorls I/II/III/IV/V with 9/8/7/5/5 chaetae respectively (Fig. 12A, B). Leg II: coxa with one chaeta; trochanter with two chaetae; femur with 10 chaetae; tibiotarsus with 34 chaetae and two oval organs (**O2pe**, **O2ae**), whorls I/II/III/IV/V with 9/8/7/5/5 chaetae, respectively (Fig. 12C, D). Leg III: coxa with two chaetae; trochanter with four chaetae; femur with 11 chaetae, one as a short curved chaeta; tibiotarsus in males with 36 chaetae and two oval organs (**O2pe**, **O2ae**), chaeta IIpe leaf-shaped in a papilla plus two regular chaetae, whorls I/II/III/IV/V with 9/9/7/6/5 chaetae respectively (Fig. 12E, F); tibiotarsus in females with 35 chaetae and two oval organs (**O2pe**, **O2ae**), chaetae **Ipi**, **IIpi**, **IIIpi** and **IVpi** serrated, whorls I/II/III/IV/V with 9/8/7/6/5 chaetae respectively (Fig. 12G). Oval organs of all tibiotarsi in both sexes with a tiny inner sensillum, each (not represented in the figures). Foot complexes: pretarsi I–III with anterior and posterior chaetae each; ungues I–II more slender than the unguis III, with one dorsal unpaired tooth and one internal unpaired tooth, ungues without tunica and pseudonychia; unguiculi I–III without teeth, with all lamellae smooth, unguiculi filament pre-apical and reaching the apex of the ungues in legs I and II, absent in leg III; ratio of ungues I–III in the holotype = 1:0.97:0.96.

#### Etymology.

The species was named after its type locality, Piauí State, Brazil.

#### Remarks.

*Sphaeridiapiauiensis* sp. nov. belongs to the *irmleri* group sensu Bretfeld and Gauer (1994) due to its complex male ventral tube without asymmetrical structures or medial processes. The *irmleri* group holds only three Neotropical species: *S.irmleri* Bretfeld & Gauer, 1994, *S.fibulifera* Bretfeld & Gauer, 1994, and *S.peruensis* Bretfeld & Schulz, 2012. Although they resemble *S.piauiensis* sp. nov. by the complex male ventral tube, *S.irmleri* and *S.fibulifera* males have a smooth and long chaeta **IIpe** on tibiotarsus III, while males of *S.piauiensis* sp. nov. and *S.peruensis* have a leaf-shaped **IIpe** chaeta. Regarding the tibiotarsal chaetotaxy, *S.piauiensis* sp. nov. differs from *S.peruensis* by presenting the tibiotarsus III **IIIpi** chaeta of normal shape in males and ventral tube with 1+1 chaetae, while *S.peruensis* presents tibiotarsus III **IIIpi** chaeta forked in males and the absence of ventral tube chaeta. Also *S.peruensis* presents two pairs of hooks on the furca (a pair on the manubrium and another on the dens), while they are absent in *S.piauiensis* sp. nov. The leaf-shaped chaeta on the tibiotarsus III of the male is also seen in the Brazilian species *S.heloisae* Arlé, 1984, but this species belongs to the *brevipila* group due to the presence of a posterior medial process on the male ventral tube, absent in *S.piauiensis* sp. nov. The female of *S.piauiensis* sp. nov. presents the tibiotarsus III chaeta **Ipi** toothed, which makes it the sole species from the Neotropical Region with such recorded morphology. Additionally, the new species does not present the chaeta **p0** in the labral region (Fig. 8C), something unusual to other Symphypleona genera. However, this information is mostly undescribed for other species of *Sphaeridia*, so it is not clear if it has any value as a specific diagnostic trait.

### ﻿Identification key (based on males) and distribution of Neotropical species of Sphaeridia*

**Table:** 

1	Ventral tube modified, with complicated structures and/or with a posterior medial process	**2**
–	Ventral tube without modifications or only with a pair of vesicles (*pumilis* group)	**4**
2	Ventral tube with complicated asymmetrical structures and/or with a posterior medial process	**3**
–	Ventral tube without a medial process, with posterior and lateral complicated symmetrical structures (*irmleri* group)	**14**
3	Ventral tube without medial process, with complicated asymmetrical structures (*spira* group)	**17**
–	Ventral tube with a posterior medial process (*brevipila* group)	**18**
4	Ventral tube without posterior vesicles	**5**
–	Ventral tube with 1+1 posterior vesicles	**6**
5	Chaeta **IIpe** on tibiotarsus III as a bipartite blade	***S.tschirnhausi* Bretfeld & Schulz, 2012** (Peru)
–	Chaeta **IIpe** on tibiotarsus III as a short leaf-shaped or normal chaeta on papilla	***S.betschi* Arlé, 1984** (Brazil)
6	Chaeta **IIpe** on tibiotarsus III as a bipartite blade	**7**
–	Chaeta **IIpe** on tibiotarsus III otherwise	**8**
7	Male color grey-blue, dorsal dens with 16 chaetae, ventral tube anteriorly with 1+1 small processes (Fig. 4E, F)	***S.squamifera* Bretfeld & Gauer, 1994** (Brazil)
–	Male color blue-violet, dorsal dens with 17 chaetae, ventral tube anteriorly without processes	***S.denisi* Massoud & Delamare-Deboutteville, 1964** (Peru)
8	Chaeta **IIpe** on tibiotarsus III forked	***S.aserrata* Mari Mutt, 1987** (Colombia)
–	Chaeta **IIpe** on tibiotarsus III otherwise	**9**
9	Chaetae **IIIpi** and **IVpi** on tibiotarsus III of normal shape or thin	**10**
–	Chaetae **IIIpi** and **IVpi** on tibiotarsus III toothed	**11**
10	Chaeta **IIpe** on tibiotarsus III long and thin, mucro	***S.neopumilis* Bretfeld & Gauer, 1994** (Colombia)
–	Chaeta **IIpe** on tibiotarsus III long and thick, mucro long	***S.pumilis* Krausbauer, 1898** ** (Algeria, Argentina, Australia, Brazil, Chile, Costa Rica, Cuba, Germany, Italy, Jamaica, Mexico, Peru, Russia, Suriname, Sweden, and Venezuela)
11	Chaeta **IIpe** on tibiotarsus III long, ventral tube anteriorly with 1+1 small projections (Fig. 2E)	***S.coronata* Bretfeld & Gauer, 1994** (Brazil)
–	Chaeta **IIpe** on tibiotarsus III otherwise, ventral tube anteriorly without modifications	**12**
12	Frontal head chaetae long and thick, chaeta **IIpe** on tibiotarsus III smooth and thick, on papilla	***S.robusta* Bretfeld & Gauer, 1994** (Brazil)
–	Frontal head chaeta long or thick, chaeta **IIpe** on tibiotarsus III normal	**13**
13	Color pale, frontal head chaetae thick	***S.clara* Bretfeld & Gauer, 1994** (Brazil)
–	Large abdomen dorsally blue or completely blue with a lateral band blue/brownish/grey or white, frontal head chaetae long	***S.pilleata* Bretfeld & Gauer, 1994** (Brazil)
14	Chaeta **IIpe** on tibiotarsus III leaf-shaped	**15**
–	Chaeta **IIpe** on tibiotarsus III smooth and long	**16**
15	Tibiotarsus III chaeta **IIIpi** forked, ventral tube without chaetae (Fig. 4A, B), manubrium and dens with a pair of hooks	***S.peruensis* Bretfeld & Schulz, 2012** (Peru)
–	Chaetae **IIIpi** of normal shape, ventral tube with 1+1 chaetae (Fig. 11A), manubrium and dens without hooks (Fig. 11B, C)	***S.piauiensis* Medeiros & Bellini, sp. nov.** (Brazil)
16	Tibiotarsus III chaeta **IVpi** toothed, dens basal appendage present, ventral tube with 1+1 chaetae (Fig. 2G, H)	***S.fibulifera* Bretfeld & Gauer, 1994** (Brazil)
–	Tibiotarsus III chaeta **IVpi** normal, dens basal appendage absent, ventral tube without chaetae (Fig. 3C)	***S.irmleri* Bretfeld & Gauer, 1994** (Brazil)
17	Ventral tube posteriorly with asymmetrical membrane and without chaetae (Fig. 4G, H)	***S.spira* Bretfeld & Gauer, 1994** (Colombia)
–	Ventral tube posteriorly with 3–5 finger-like medial processes, 1+1 medial asymmetrical processes, 1+1 lateral thin processes and 1+1 chaetae (Fig. 4I, J)	***S.sturmi* Bretfeld & Gauer, 1994** (Colombia)
18	Chaeta **IIIpi** toothed or forked	**19**
–	Chaeta **IIIpi** smooth with modifications or as a normal chaeta	**29**
19	Medial process of the ventral tube asymmetrical, with truncate apexes or knobbed at the apex (Fig. 1C)	***S.biclava* Bretfeld & Trinklein, 2000** (Ecuador)
–	Medial process of the ventral tube otherwise	**20**
20	Chaeta **IVpi** smooth with modifications or as a normal chaeta	**21**
–	Chaeta **IVpi** toothed	**24**
21	Ventral tube posteriorly with 1 median forked process, 5+5 processes (2+2 as spines, 1+1 with a irregular tip and 2+2 blunt structures) (Fig. 2F)	***S.decemdigitata* Bretfeld & Schulz, 2012** (Peru)
–	Ventral tube otherwise	**22**
22	Ventral tube without chaeta (Fig. 3H)	***S.panguanae* Bretfeld & Schulz, 2012** (Peru)
–	Ventral tube with 1+1 chaetae	**23**
23	Large abdomen with two broad blue stripes, Ant III–IV blue, tibiotarsus III chaeta **IIpe** smooth, long, and acuminated, **IVpi** smooth and thick, ventral tube with 1 short blunt median process, 1+1 processes with 3 teeth each, plus several small processes (Fig. 1D)	***S.bivirgata* Bretfeld, 2002** (Brazil)
–	Color pale with a large black spot covering the dorsal part of the large abdomen, tibiotarsus III chaetae **IIpe** and **IVpi** normal, ventral tube posteriorly with duck-shaped process (Fig. 1J)	***S.carioca* Arlé, 1984** (Brazil)
24	Chaeta **IIpe** leaf-shaped or vesicle-like, or spiniform on papilla	**25**
–	Chaeta **IIpe** smooth and long, or thin, or as a normal chaeta	**26**
25	Head and body with small blue spots, tibiotarsus III chaeta **IIpe** leaf-shaped or vesicle-like, posterior ventral tube with 1 blunt median process and 1+1 irregular distal processes (Figs 4K, 5A)	***S.torifera* Bretfeld & Schulz, 2012** (Peru)
–	Specimens pale, tibiotarsus III chaeta **IIpe** spiniform on papilla, posterior ventral tube with 1 median finger-shaped process and 1+1 roundish processes (Fig. 3I, J)	***S.paroara* Arlé, 1984** (Brazil)
26	Ventral tube posteriorly with 1 medial process bifurcated at the apex (Fig. 1K)	***S.catapulta* Bretfeld & Gauer, 1994** (Colombia)
–	Ventral tube posteriorly otherwise	**27**
27	Tibiotarsus III chaeta **IIpe** normal, ventral tube posteriorly with 1 median process with acutely truncate blunt apex and 1+1 lateral acuminated processes	***S.pippetti* Murphy, 1965** (Peru)
–	Tibiotarsus III chaeta **IIpe** smooth and long, ventral tube otherwise	**28**
28	Color pale blue or brownish, ventral tube posteriorly with 1 thick medial process with a broad tip and 1+1 lateral curved processes (Fig. 2K, L)	***S.franklinae* Bretfeld & Gauer, 1994** (Brazil)
–	Specimens with violet median and horizontal bands, antennae, and legs blue, furca pale, ventral tube posteriorly with 1 short median process, 1+1 slender lateral processes, 1+1 blunt tridentate lateral processes, with a thin, protruding membrane in a hand-glass shape (Fig. 3E, F)	***S.mandibulata* Bretfeld & Gauer, 1994** (Colombia)
29	Chaeta **IIpe** leaf-shaped, ventral tube posteriorly with 1 medial, 1+1 hook-like, 1+1 acuminate and 1+1 blunt processes (Fig. 3A, B)	***S.heloisae*Arlé 1984** (Brazil)
–	Chaeta **IIpe** and ventral tube otherwise	**30**
30	Ventral tube without chaetae	**31**
–	Ventral tube with 1+1 chaetae	**32**
31	Specimens entirely blue, or with paler dorsal sides, ventral tube posteriorly with 1 thick and blunt medial process and 2+2 pointed processes (vampire-like) (Fig. 5F–H)	***S.vampyra* Bretfeld & Schulz, 2012** (Peru)
–	Body diffusely pigmented, ventral tube posteriorly with 1+1 ring-shaped posterior processes (Fig. 2I, J)	***S.fluminensis* Arlé, 1984** (Brazil)
32	Tibiotarsus III chaeta **IVpi** toothed, ventral tube posteriorly with 1 median bladder-like process, 1+1 finger-like spines and 1+1 irregular lobes (Fig. 5B–D)	***S.tropica* Bretfeld & Schulz, 2012** (Peru)
–	Tibiotarsus III chaeta **IVpi** and ventral tube otherwise	**33**
33	Ventral tube with 2 median processes (Figs 2C, D, 4D, 5I)	**34**
–	Ventral tube otherwise	**36**
34	Color blue, laterally dark, ventral tube with 1+1 middle and 1+1 short, blunt lateral processes (Fig. 2C, D)	***S.chisacae* Bretfeld & Gauer, 1994** (Colombia)
–	Color pattern and ventral tube otherwise	**35**
35	Ventral tube posteriorly with 2 median posterior processes, the anterior with 1 posterior tooth, plus 1+1 small lateral processes (Fig. 5I)	***S.winteri* Massoud & Delamare Deboutteville, 1964** (Peru)
–	Ventral tube with 2 median posterior processes, the anterior one with 2 teeth, plus 1+1 lateral regular processes (Fig. 4D)	***S.schalleri* Massoud & Delamare-Deboutteville, 1964** (Peru)
36	Ventral tube anteriorly with 1+1 mandible-like processes laterally pointed (Fig. 1E–G)	***S.boettgeri* Bretfeld & Gauer, 1994** (Paraguay)
–	Ventral tube anteriorly otherwise	**37**
37	Ventral tube anteriorly without any clear process (Fig. 3D)	***S.lobata* Bretfeld & Gauer, 1994** (Colombia)
–	Ventral tube anteriorly with processes	**38**
38	Specimens dark blue, ventral tube anteriorly with 1+1 bifurcated lateral processes (Figs 1L, 2A, B)	***S.cerastes* Bretfeld & Gauer, 1994** (Brazil)
–	Specimens purplish, ventral tube anteriorly with at least 3+3 processes (Fig. 1H, I)	***S.cardosi* Arlé, 1984** (Brazil)

* Here we did not include the species inquirendae. Further details on the male ventral tube are presented in Figs 1–5 and 11A.

** *S.pumilis*, following Bretfeld (1995: 20), is a Holarctic species, and its registers from others localities are likely from the *pumilis* complex of species. Even so, we maintain the species in the key to represent the species found in many countries of the Neotropical Region.

### ﻿Genus Denisiella

Taxon classificationAnimaliaCollembolaSminthurididae

Folsom & Mills, 1938

52FD4C82-2688-5A88-9BE5-E7E1832EF46C

#### Diagnosis of the genus.

Males with highly dimorphic antennae, Ant II with **Tra1–2**, **b1–b7** elements, **b1–b6** together, **b7** isolated, Ant III with **Tra3** as a bothriotrichum or a regular chaeta, elements **c1** and **c3** always present. Ant IV undivided in both sexes, usually with blunt sensilla. Eyes 6+6 to 8+8. Th III and large abdomen in males without vesicles. Bothriotricha **ABC** misaligned. Posterior large abdomen with or without long chaetae. Ventral tube without modifications. Each anal valve with 0–2 barbulated spines in both sexes. Tibiotarsus I proximal organ usually present in males, formed by four modified sensilla. Tibiotarsus II with or without a polycarinate chaeta. Tibiotarsus III with 0–5 serrated spines in both sexes. Distal tibiotarsal organ on leg III absent. Dens lacking spine-like chaetae. Mucro narrow, inner edge serrated, outer smooth, mucronal chaeta present (adapted and revised from Börner 1908; Denis 1925; Denis 1931; Folsom 1932; Snider 1988; Palacios-Vargas 1995; Palacios-Vargas and Bernava 1999; Palacios-Vargas 2007; Ospina and Palacios-Vargas 2009; Schulz and van Harten 2013; Palacios-Vargas et al. 2018).

#### Type species.

*Sminthuridesseurati* Denis, 1925.

#### Distribution.

Americas, Africa, and Indo-Asia (Bellinger et al. 1996–2023).

#### Remarks.

**Figure 13.**
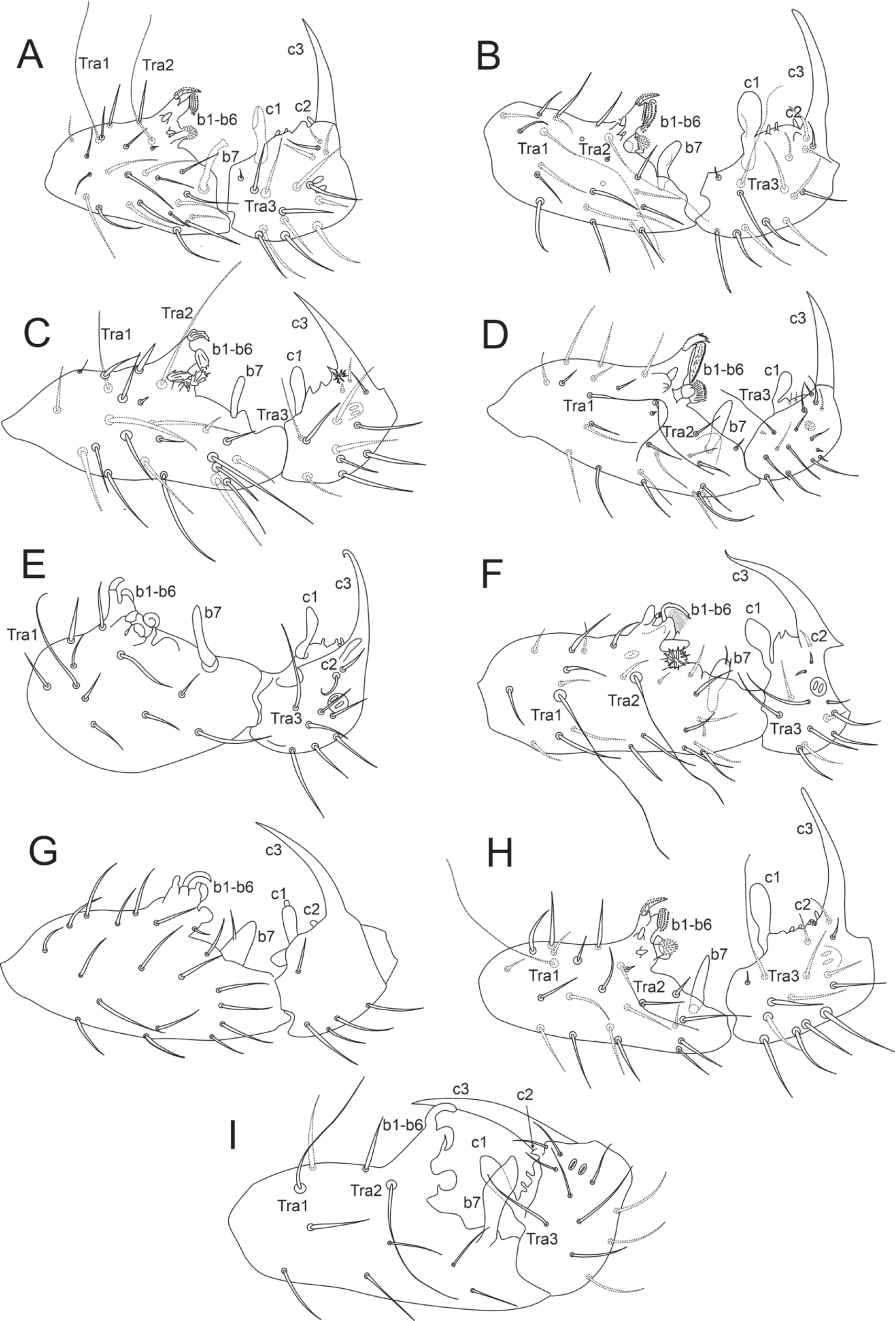
Chaetotaxy of Ant II and III of males of *Denisiella***A***D.betschi* Palacios-Vargas, Ferreira & Zeppelini, 2018 **B***D.caatingae* Palacios-Vargas, Ferreira & Zeppelini, 2018 **C***D.colombiana* Ospina & Palacios-Vargas, 2009 **D***D.diomedesi* Palacios-Vargas, 2007 **E***D.lithophila* Snider, 1988 **F***D.maesorum* Palacios-Vargas, 1995 **G***D.ramosa* (Folsom, 1932) **H***D.rhizophorae* Palacios-Vargas, Ferreira & Zeppelini, 2018 **I***D.seurati* (Denis, 1925) (ventral view). Figures adapted from species’ original descriptions.

*Denisiella* species arguably have the most sexually dimorphic antennae among all the Sminthurididae, with several modified elements on the male claspers (Massoud and Betsch 1972; Betsch 1980; Medeiros et al. 2022). The morphology of such elements varies between the species (Fig. 13), but even so, most of them are present, with the exception of the **c2** element on Ant III, which may be absent in some species (Börner 1908; Denis 1931; Palacios-Vargas and Bernava 1999; Schulz and van Harten 2013), and **Tra3** on Ant III, which interchanges between a regular chaeta or a bothriotrichum (Denis 1925; Palacios-Vargas 1995; Palacios-Vargas et al. 2018) (Table 2).

**Table 2.** Main diagnostic characters of *Denisiella* species.

Species (known sexes) /characters	Color (♂)	Color (♀)	Ant III c2 element (♂)	Ant III Tra element (♂)	Ant II spiniform chaetae (♀)	Ant III spiniform chaetae (♀)	Ant IV sensilla (♂)	Eyes	Frontal head chaetae (♂)	Nasal organ (♂)	Posterior large abdomen with long chaetae	Barbulated spines on dorsal anal valve (ps1–2) (♂)	Barbulated spines on ventral anal valves (pi1–3) (♂)	Barbulated spines on dorsal anal valve (ps1–2) (♀)	Barbulated spines on ventral anal valves (pi1–3) (♀)	Proximal tibiotarsal organ leg I (♂)	Polycarinate chaeta on tibiotarsus II	Modified spines on tibiotarsi I–III	Ungual inner tooth on leg I	Ungual inner tooth on leg III	Dens dorsal chaetae (♂)	Dens ventral chaetae formula
*D.betschi*^11^ (♂)	?	?	+	Rs	?	?	3	6+6	Sf, Ac, Ba	-	?	-	-	?	?	Cpf, Rg	+	0/0/4 (♂)	+	+	37	3,3,3,3,2,1,1,1
*D.bretfeldi*^10^ (♂,♀)	Body violet with legs, antennae and furcula yellowish	Body with broad violet edges, middle yellowish, legs, antennae and furcula slightly violet	?	?	?	?	2	6+6	?	+	-	?	?	-	**pi1**	Bd	-	0/0/4? (♂)	+	+	30	3,2,1,1,1,1,1 (♀) 3,3,3(2),2(1),2(1),1,1,1,1 (♂)
*D.caatingae*^11^ (♂)	?	?	+	Bo	?	?	5	6+6	Ac, Lg	+	?	?	?	?	?	Sd	+	0/0/4 (♂)	+	+	44	3,3,3,3,2,2,2,1,1,1
*D.colombiana*^9^ (♂,♀)	Body dark purple and furcula almost transparent, legs and Ant with purple pigment at their bases	Body dark purple, legs, antennae and furcula pale purple	-	Rs	+	+?	?	6+6	Ac, Sf	-	-	-	**pi1, pi3**	**ps2**	**pi1, pi3**	Bd	+	0/0/4 (♂)	+?	+	42	3,3,3,3,2,1,1,1
*D.diomedesi*^8^ (♂)	Body and antennae purple, legs and furcula pale, with small purple pigment at their bases.	?	-	Bo	?	?	1	6+6	Ac, Sf, Lg	+	?	-	-	?	?	Cpf, Rg	+	0/0/4 (♂)	+	+	42	3,3,3,2,2,1,1,1
*D.lithophila*^5^ (♂,♀)	Head and body with cream white and blue mosaics	Head and body blue-black with cream markings in irregular mosaics	+	Rs	-	-	?	6+6	?	-	- (♀), + (♂)	-	**pi3**	-	-	-	-	0/0/3 (♂)	+	+	46	3,3,3,3,2,2,1,1?
*D.maesorum*^6^ (♂,♀)	Body, legs and antennae purple	Head and body mostly purple	+	Rs	-	-	+	6+6	Sf, Rg	-	+?	-	-	-	-	Cpf, Rg	-	1/1/4 (♂,♀)	+	+	46	3,3,3,3,2,1,1,1
*D.nayarita*^7^ (♀)	?	Trunk dorsally pigmented, appendages slightly tinged with purple	?	?	+	+	?	6+6	Ac, Sf	?	+	?	?	**ps2**	**pi1, pi3**	?	?	0/0/5 (♀)	+	+	38	3,3,3,3,2,1,1,1,1?
*D.ramosa*^4^ (♂,♀)	Body, legs and antennae purple	Head and body mostly purple	+	?	-	-	?	8+8*	Ac, Sf	-	?	-?	-?	**ps2**	**pi1**	Rgd	?	?	+	+	?	?
*D.rhizophorae*^11^ (♂,♀)	?	?	+	Bo	-	-	6	6+6	Ac, Lg	-	?	-	-	-	2+2	Rgd	+	0/0/4 (♂)	+	-	38	3,3,3,3,2,1,1,1
*D.seurati*^2^ (♂,♀)	Pale and Pale violet	Cream head and body with parts of the large abdomen, appendages and antennae purple	+	Rs	-	-	3?	8+8	?	-	?	?	?	-	1+1	Rgd	-	1/0/5 (♂)	+	+	35?	?
*D.serroseta*^1,10^** (♂,♀)	Head and body dark purple (not intense)	Head and body dark purple	?	?	+	+	?	6+6	Sh, Sf	-?	+	?	?	?	?	?	-?	+(♂)	+	+	?	?
*D.sexpinnata*^3^(♀)	?	Head and body mostly violet	?	?	?	?	?	8+8	?	?	+	?	?	**ps2**	**pi1, pi3**	?	?	1/0/5 (♀)	+	+	37	?
*D.piracurucaensis* sp. nov. (♂,♀)	Purple with pale appendages	Not observed	+	Rs	+	+	19	8+8	Ac, Sf	-	+	**ps2**	**pi1**	**ps2**	**pi1, pi3**	Cpf	-	1/0/4 (♂,♀)	+	+	46	3,3,3,3,2,1,1(0),1

Data based in: ^1^Börner (1908); ^2^Denis (1925); ^3^Denis (1931); ^4^Folsom (1932); ^5^Snider (1988); ^6^Palacios-Vargas (1995); ^7^Palacios-Vargas and Bernava (1999); ^8^Palacios-Vargas (2007); ^9^Ospina and Palacios-Vargas (2009); ^10^Schulz and van Harten (2013); ^11^Palacios-Vargas et al. (2018). Legends: + = present; - = absent; ? = unclear/unknown; Ac = Acuminate; Ba = Barbulated spine; Bd = Big dome; Bo = Bothriotricha; Cpf = Campaniform; Lg = Long; Rg = rugose; Rgd = Rhagidial; Rs = Regular shape; Sd = Small dome; Sf = Spiniform; Sh = Short; * = Folsom (1932) remarked the species has at least 12 eyes, but possible 16, so we are considering 8+8 eyes; ** = we considered *D.serroseta* presents serrated spines on tibiotarsus III and an internal tooth on unguis III following Börner (1908: 58–60, figs 5, 9, 11) description, differently from the information in Palacios-Vargas et al. (2018: 123, table 1). A detailed view of the male antennal clasper is presented in Figs 13 and 15B, C.

In the same way as to many other Sminthurididae, including *Sphaeridia*, the diagnosis and comparisons between *Denisiella* taxa is mostly based on males’ morphology. Because of this, some *Denisiella* descriptions lack data on the females’ morphology, especially regarding the antennal and abdominal chaetotaxy (Börner 1908; Denis 1931; Palacios-Vargas 2007; Schulz and van Harten 2013; Palacios-Vargas et al. 2018). In our survey we observed *D.nayarita* Palacios-Vargas and Bernava, 1999 and *D.sexpinnata* (Denis, 1931) were described based only on females, while their males’ morphology is completely unknown. So, we suggest both taxa as *species inquirendae*, as their identities are not fully clear and it is not possible to clearly distinguish them from other taxa, especially from those described based only on male morphology. In a similar way, *D.serroseta* (Börner, 1908) description lacks information about the male antennae, small and large abdomen of both sexes, and shows unclear data on the chaetotaxy of legs and furca. In this scenario this description does not fit the current taxonomy of Sminthurididae and does not allow us to clearly separate the species from its congeners; therefore, we also suggest *D.serroseta* as a *species inquirenda*.

Some species of *Denisiella* can be readily distinguished from several others by the presence/absence of a unique feature located between de clypeal and interantennal areas of male head, the nasal organ (Palacios-Vargas et al. 2018). Since this structure is formed by strongly modified projections, we believe it may have a phylogenetical significance and probably points to different lineages within the genus. So here we tentatively separate the genus in two distinct groups: the *seurati* group, for the species without the nasal organ, holding: *D.betschi* Palacios-Vargas, Ferreira & Zeppelini, 2018, *D.colombiana* Ospina & Palacios-Vargas, 2009, *D.lithophila* Snider, 1988, *D.maesorum* Palacios-Vargas, 1995, *D.ramosa* Folsom, 1932, *D.rhizophorae* Palacios-Vargas, Ferreira & Zeppelini, 2018, *D.seurati* Denis, 1925, *D.serroseta* Börner, 1908 and *D.piracurucaensis* sp. nov.; and the *diomedesi* group, for the species with the nasal organ, with the species: *D.bretfeldi*, Schulz & van Harten, 2013, *D.caatingae* Palacios-Vargas, Ferreira & Zeppelini, 2018 and *D.diomedesi* Palacios-Vargas, 2007.

### ﻿ Denisiella piracurucaensis

Taxon classificationAnimaliaCollembolaSminthurididae

Silva, Medeiros & Bellini sp. nov.

18630065-BE96-5A79-B8B7-5AED4A8D9A99

https://zoobank.org/69CFC9F2-DEF3-49DD-B7D1-8DF387F315B1

**Figure 14.**
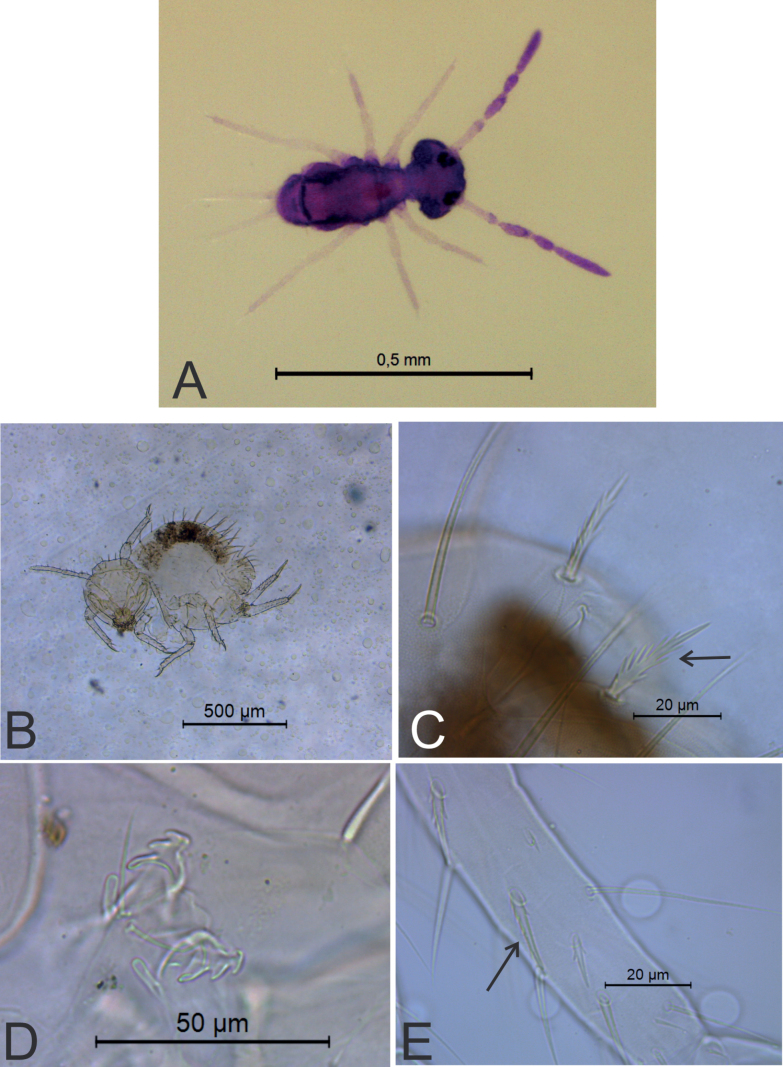
*Denisiellapiracurucaensis* sp. nov. photographs **A** habitus of a male in ethanol (dorsal view) **B** habitus of a female in slide (lateral view) **C** barbulate chaeta in the small abdomen of a female **D** tenaculum of a female **E** serrate chaeta on the tibiotarsus III of a male.

**Figure 15.**
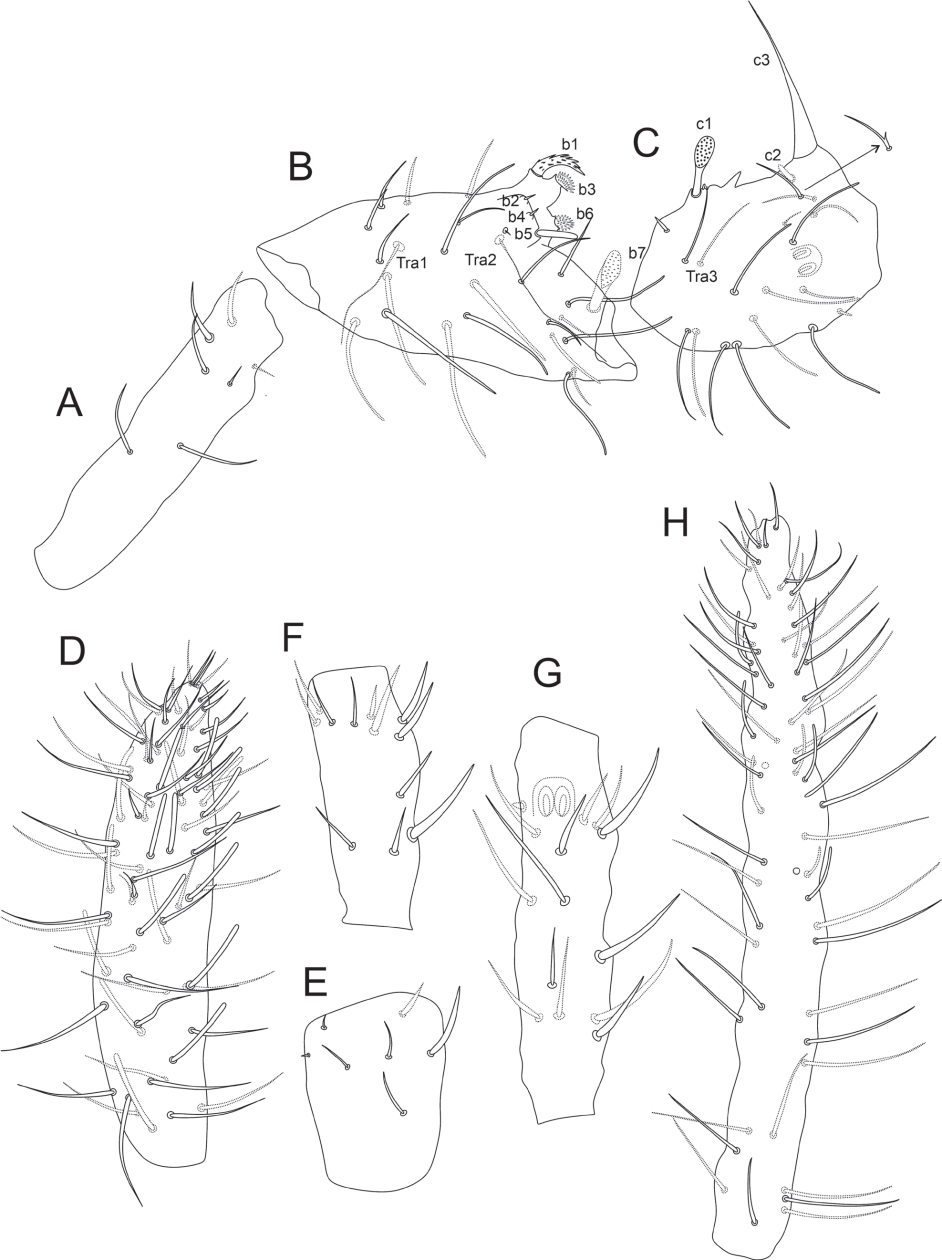
*Denisiellapiracurucaensis* sp. nov. antenna (dorsal view) **A**–**D** male antenna **A**Ant I **B**Ant II **C**Ant III **D**Ant IV **E**–**H** female antenna **E**Ant I **F**Ant II **G**Ant III **H**Ant IV.

**Figure 16.**
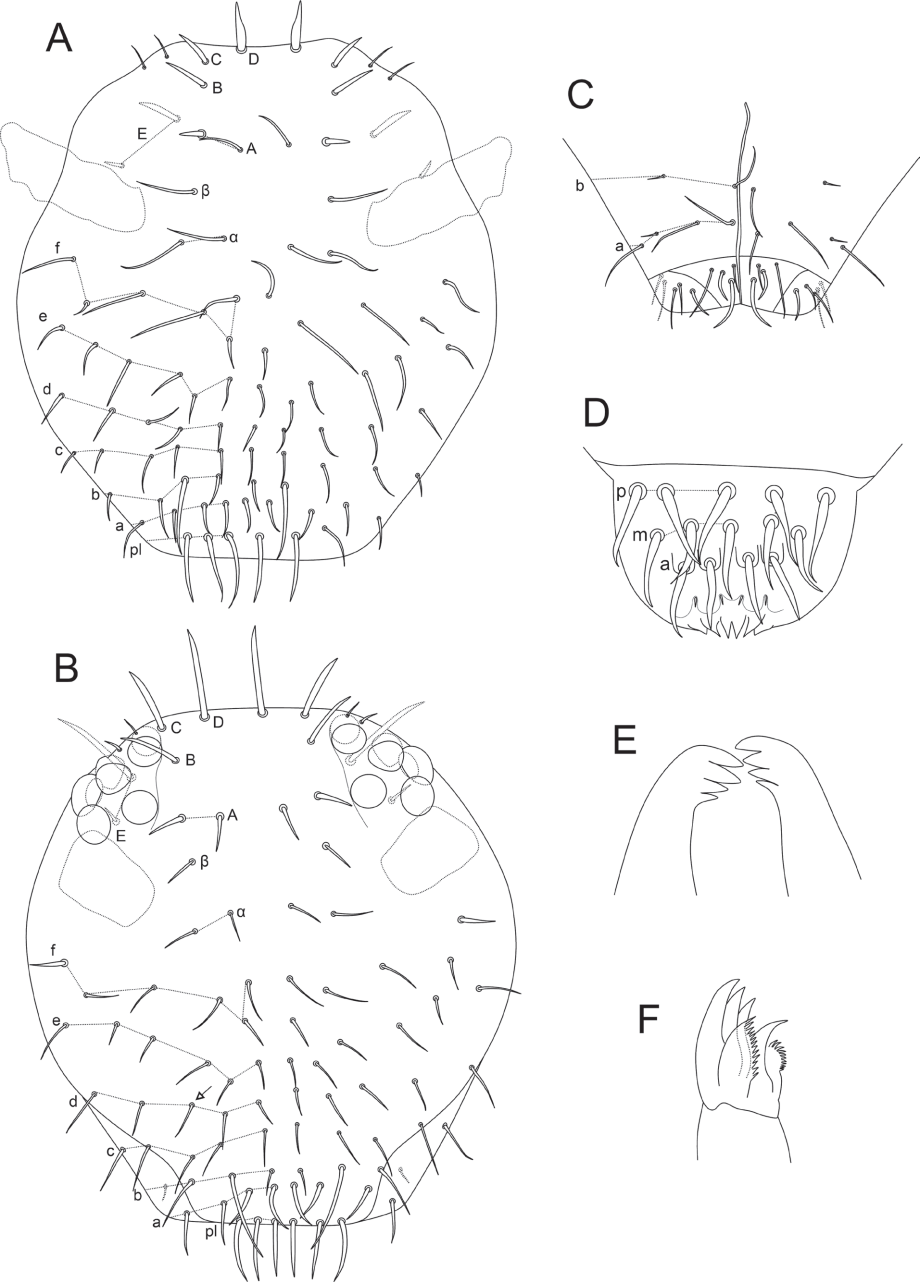
*Denisiellapiracurucaensis* sp. nov. head **A** male anterior head cheatotaxy (eyes omitted) **B** female anterior head cheatotaxy, arrow indicates a chaeta which can be present or absent **C** male labial and post-labial (ventral) chaetotaxy **D** male labral chaetotaxy **E** female mandibles apexes (incisive teeth) **F** female left maxilla capitulum.

**Figure 17.**
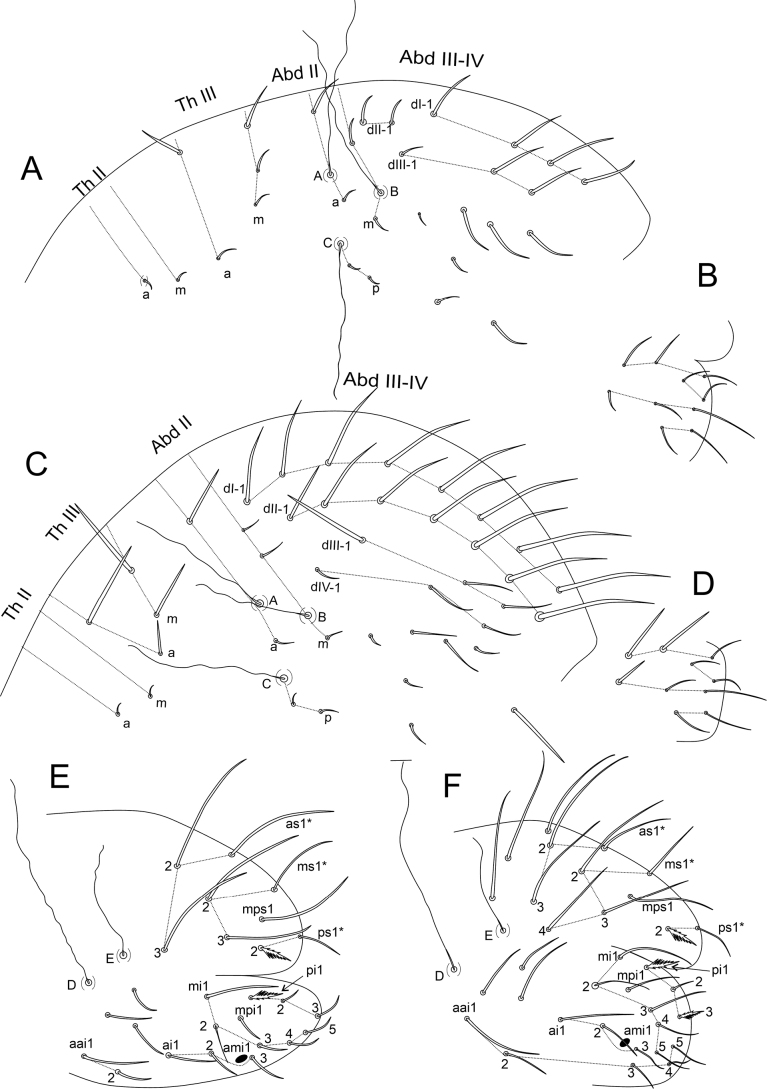
*Denisiellapiracurucaensis* sp. nov. trunk **A** male large abdomen **B** male parafurcal area **C** female large abdomen **D** female parafurcal area **E** male small abdomen **F** female small abdomen.

**Figure 18.**
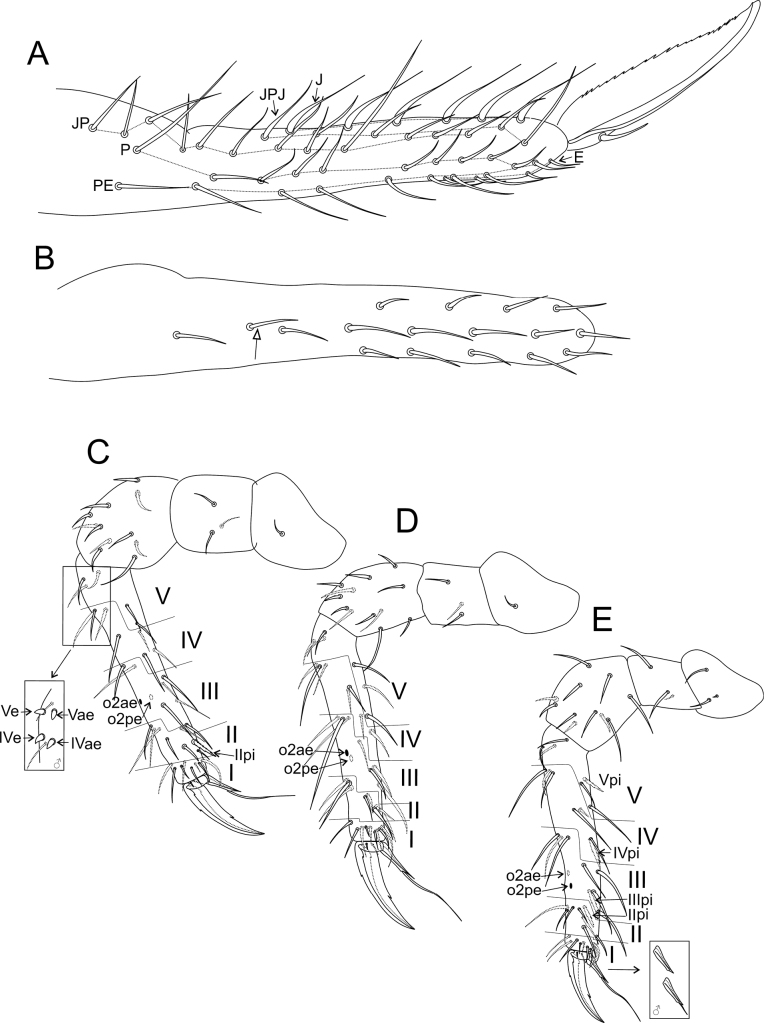
*Denisiellapiracurucaensis* sp. nov. trunk appendages **A** female dorsal dens chaetotaxy and mucro **B** female dorsal dens chaetotaxy, arrow indicates a chaeta which can be present or absent **C** female coxa, trochanter, femur, tibiotarsus and empodial complex of leg I, detail shows the campaniform sensilla of males **D** female coxa, trochanter, femur, tibiotarsus and empodial complex of leg II **E** female coxa, trochanter, femur, tibiotarsus and empodial complex of leg III, detail shows the unguiculus filament (present or absent) of males.

Figs 14 , 15 , 16 , 17 , 18 , Table 2

#### Type material.

***Holotype***: male on slide, Brazil, Piauí state, Piracuruca municipality, Sete Cidades National Park, ‘Primeira Cidade’ (4°05'42.53"S, 41°40'50.7"W), 168 m, in sandy soil, ecotonal zone between Caatinga and Cerrado biomes, 14/V/2021, A.M.N. Silva col., pitfall traps. ***Paratypes*** three males and four females on slides.

#### Diagnosis.

Male head and trunk uniformly dark purplish, legs, furca and antennal bases pale, distal Ant I and Ant II–IV purplish (Fig. 14A). Male Ant II with 11, Ant III with seven long blunt sensilla, respectively, **Tra3** as a regular chaeta, Ant IV with 19 sensilla. Female Ant II and Ant III with spiniform chaetae, Ant IV with eight sensilla. Eyes 8+8. Frontal head with acuminate spiniform chaetae, longer in the females (Fig. 14B). Nasal organ absent. Female anal valves with **ps2**, **pi1** and **pi3** as barbulated chaetae (Fig. 14C). Parafurcal area with 10 chaetae in both sexes. Each tenaculum ramus with three teeth, corpus with 1+1 chaetae (Fig. 14D). Males with four campaniform chaetae on the proximal tibiotarsal organ in leg I. Polycarinate chaeta on tibiotarsus II absent. Tibiotarsus III chaetae **IIpi**, **IIIpi**, **IVpi**, and Vpi serrated (Fig. 14E). Dorsal dens **J** chaetae enlarged at their bases, somewhat spiniform, ventral chaetotaxy formula from the apex to the basis as 3,3,3,3,2,1,1,1 or 3,3,3,3,2,1…1.

#### Description.

***Body*** (head + trunk) length of the type series ranging between 0.41 and 1.4 µm, holotype with 0.5 µm, male average size = 0.52 µm, females average size = 1.1 µm, entire type series average size = 0.85 µm. Male head and trunk uniformly dark purplish, legs, furca and antennal bases pale, distal Ant I and Ant II–IV purplish (Fig. 14A). Female color pattern unknown (studied specimens already on glass slides).

***Head*** (Figs 15–16). Antennae length: 0.32 µm in the holotype. Holotype antennal segments ratio I:II:III:IV as 1.0:1.1:0.5:1.6. Male antenna: Ant I elongated with seven chaetae, two smaller at the apex, two other apical chaetae thicker than the others (Fig. 15A). Ant II with 11 regular chaetae, 11 long blunt sensilla, two bothriotricha (**Tra1**, **Tra2**), one microsensillum, modified chaetae **b1**, **b3** and **b6** on the anterior tubercle, **b2**, **b4** and **b5** on the posterior tubercle, **b7** thick, apically flattened and granulated (Fig. 15B). Ant III with eight regular chaetae, one of them as a bifurcated chaeta in one antenna of a single specimen, seven long blunt sensilla, three microsensilla, **Tra3** as a regular chaeta, apical organ sensory rods in one single shallow invagination, modified chaetae **c1**–**3** present, **c1** thick, apically flattened, and grainy, **c2** as a small blunt projection and **c3** as a thick smooth spine in a papilla with at least one cuticular spine between **c1** and **c3** (Fig. 15C). Ant IV with ~ 58 regular chaetae, plus 19 blunt sensilla (Fig. 15D). Female antenna: Ant I short with seven chaetae, two smaller at the apex, two other apical chaetae thicker than the others (Fig. 15E). Ant II with 12 chaetae (Fig. 15F), five of them regular, seven somewhat spiniform. Ant III with 13 chaetae, six of them somewhat spiniform, plus one apical microsensillum; apical organ sensory rods in a single invagination (Fig. 15G). Ant IV longer than Ant III, with ~ 52 regular chaetae, plus eight blunt sensilla (Fig. 15H). Head capsule (both sexes): Eyes 8+8, with two interocular chaetae, head capsule normal (not elongated) (Fig. 16A, B). Clypeal area **a–f** lines with 8/6/5/4–5/6/6 chaetae, respectively. Interantennal area **α** and **β** lines with 2/1 chaetae, respectively; frontal area **A–E** lines with 2/1/1/1/2 chaetae, respectively, frontal spiniform chaetae of females longer than in the males (Fig. 16A–C). Labial basomedian field with four chaetae, basolateral field with five (Fig. 16C). Six prelabral chaetae long and thick (Fig. 16A, B); labral **p**, **m**, and **a** lines with 5/5/4 thick chaetae, respectively; each **a** line chaeta in a single papilla; labral papillae present as small spines, labral apex cuticle with six pointed projections (Fig. 16D). Mandibles normal (not elongated), with 4+4 incisive apical teeth (Fig. 16E). Maxillae normal (not elongated) (Fig. 16F). Labrum, labium, and mouthparts without any clear sexual dimorphism.

***Trunk*** (Fig. 17). Large abdomen: thorax continuous with the abdomen, without clear segmentations in both sexes (Fig. 17A, B). Male: Th II with one **a** and one **m** chaetae; Th III with two **a** and three **m** chaetae; Abd I without chaetae; Abd II with bothriotricha **A**, **B**, and **C** misaligned, with two **a**, two **m**, and two **p** regular chaetae; Abd III–IV with three main lines of chaetae above the bothriotrichum B: **dI–1** with four, **dII–1** with two, **dIII–1** with three chaetae respectively, plus seven chaetae below the bothriotrichum **B** (Fig. 17A). Parafurcal area in males with 10 chaetae in four rows of chaetae, with three, two, three, and two chaetae, respectively, neosminthuroid chaetae absent (Fig. 17B). Female: Th II with one **a** and one **m** chaetae; Th III with two **a** and two **m** chaetae; Abd I without chaetae; Abd II with bothriotricha **A**, **B**, and **C** misaligned, with two **a**, three **m**, and two **p** regular chaetae; Abd III–IV with four main lines of chaetae above the bothriotrichum **B**: **dI–1** with eight, **dII–1** with seven, **dIII–1** with three and **dIV–1** with three respectively, plus seven chaetae below the bothriotrichum **B**. Dorsal chaetae longer than in the males (Figs 14B, 17C). Parafurcal area in females with 10 chaetae in four rows, with three, two, three, and two chaetae, respectively, three internal somewhat spiniform (Fig. 17D). Small abdomen: including Abd V–VI in both sexes (Fig. 17E, F). Male: Abd V with bothriotricha **D** and **E**. Dorsal anal valve with **as1–3**, **ms1–3**, **mps1**, and **ps1–2** chaetae, **ps2** barbulated; **as1**, **ms1**, and ps1 unpaired; each ventral anal valve with **aai1–2**, **ai1–3**, **ami1** (as an oval organ lacking any inner sensillum), **mi1–5**, **mpi1** and **pi1–3** chaetae, **pi1** barbulated (Fig. 17E). Female: Abd V with bothriotricha **D** and **E** present. Dorsal anal valve with **as1–3**, **ms1–4**, **mps1**, and **ps1–2** chaetae, **ps2** barbulated (Fig. 17F); **as1**, ms1, and ps1 unpaired; each ventral anal valve with **aai1–5**, **ai1–3**, **ami1** (as an oval organ lacking any inner sensillum), **mi1–5**, **mpi1** and **pi1–3** chaetae present, **pi1** and **pi3** barbulated (Figs 14C, 17F). Genital plate unclear in both sexes.

***Abdominal appendages*** (Fig. 18A, B). Ventral tube with 1+1 chaetae. Each tenaculum ramus with three teeth plus an apically rounded basal appendix, corpus with 1+1 chaetae (Fig. 14D). Manubrium with 8+8 dorsal chaetae. Dens with a basal appendage and 46 dorsal (posterior) chaetae, lines **E/PE/P/JP/JPJ/J** with 4/9/10/12/5/6 chaetae, respectively; **J** line chaetae enlarged at their bases, somewhat spiniform (Fig. 18A); dens ventrally (anteriorly) with 17 chaetae, following the formula from the apex to the basis: 3,3,3,3,2,1,1,1 or 3,3,3,3,2,1…1 (Fig. 18B). Mucro narrow, with an external lamella smooth and an internal lamella serrated with ~ 22 teeth, mucronal chaeta present (Fig. 18A). Furca without any clear sexual dimorphism. Manubrium:dens:mucro ratio of the holotype = 1.0:2.5:0.76.

***Legs*** (Fig. 18C–E). Leg I: epicoxa and subcoxa without chaetae, coxa with one chaeta; trochanter with three chaetae; femur with 11 chaetae, two of them curved; tibiotarsus with 36 chaetae and two oval organs (**O2pe**, **O2ae**), whorls I/II/III/IV/V with 9/8/7/7/5 chaetae respectively, chaeta **IIpi** serrated; male proximal four chaetae **IVe**, **IVae**, **Ve**, and **Vae** modified into campaniform sensilla (Fig. 18C). Leg II: epicoxa with one chaeta, subcoxa without chaetae, coxa with one chaeta; trochanter with three chaetae; femur with 12 chaetae; tibiotarsus with 37 chaetae and two oval organs (**O2pe**, **O2ae**), whorls I/II/III/IV/V with 9/8/7/7/6 chaetae respectively (Fig. 18D). Leg III: epicoxa with one chaeta, subcoxa with one chaeta, coxa with three chaetae (two regular and one small); trochanter with three chaetae; femur with 11 chaetae, one of them curved; tibiotarsus with 38 chaetae and two oval organs (**O2pe**, **O2ae**), whorls I/II/III/IV/V with 9/8/8/7/6 chaetae respectively; chaetae **IIpi**, **IIIpi**, **IVpi**, and **Vpi** serrated (Figs 14E, 18E). Oval organs of all tibiotarsi in both sexes with a tiny inner sensillum, each (not represented in the figures). Foot complexes: pretarsi I–III with an anterior and a posterior chaetae each; ungues I–III subequal in shape, with one dorsal unpaired tooth, a pair of basal lateral teeth and one medial internal tooth; unguiculi I–III somewhat truncate, without teeth, with all lamellae smooth. Female unguiculi apical filament surpassing the apex of the ungues in legs I–III; male unguiculi apical filament surpassing the apex of the ungues in legs I and II, unguiculus III apical filament absent or reduced in leg III; ratio of ungues I–III in the holotype = 1.0:0.9:0.8.

#### Etymology.

The species was named after its type locality, Piracuruca municipality, Piauí state, Brazil.

#### Remarks.

As said before, *D.piracurucaensis* sp. nov. belongs to the *seurati* group due to the absence of the nasal organ on males. Within this group, it is more similar to *D.betschi*, *D.colombiana*, *D.maesorum* and *D.rhizophorae* due to the presence four serrated spines on the tibiotarsus III. It looks more similar to *D.colombiana* by the presence of spiniform chaetae at least on Ant II of the female and a similar ventral dens chaetotaxy. Even so, the new species can be separated from all of these taxa especially by the presence of 8+8 eyes (6+6 in the other species), **ps1** and **pi1** as barbulated chaetae on the male anal valves (both regular chaetae in *D.betschi* and *D.rhizophorae*, only **pi1** and **pi3** as barbulated chaetae in *D.colombiana*), and the absence of a polycarinate chaeta on the male tibiotarsus II (present in *D.betschi*, *D.colombiana*, and *D.rhizophorae*). Further comparisons are presented in Table 2.

### ﻿Identification key and distribution to *Denisiella* species*

**Table:** 

1	Male nasal organ present	**(*diomedesi* group) 2**
–	Male nasal organ absent	**(*seurati* group) 4**
2	Male Ant IV with two sensilla, polycarinate chaeta of tibiotarsus II absent, dens dorsally with 30 chaetae	***D.bretfeldi* Schulz & van Harten, 2013** (United Arab Emirates)
–	Male Ant IV with one or five sensilla, polycarinate chaeta of tibiotarsus II present, dens dorsally with more than 40 chaetae	**3**
3	Male Ant III **c2** element present (Fig. 13B), Ant IV with five sensilla, dens ventral chaetae formula as 3,3,3,3,2,2,2,1,1,1	***D.caatingae* Palacios-Vargas, Ferreira & Zeppelini, 2018** (Brazil)
–	Male Ant III **c2** element absent (Fig. 13D), Ant IV with one sensillum, dens ventral chaetae formula as 3,3,3,2,2,1,1,1	***D.diomedesi* Palacios-Vargas, 2007** (Panama)
4	Male proximal tibiotarsal organ of leg I campaniform	**5**
–	Male proximal tibiotarsal organ of leg I absent or otherwise	**7**
5	Eyes 8+8, modified spines of tibiotarsi I–III following the formula 1/0/4	***D.piracurucaensis* Silva, Medeiros & Bellini, sp. nov.** (Brazil)
–	Eyes 6+6, modified spines of tibiotarsi I–III formula otherwise	**6**
6	Male barbulated chaetae on frontal head present, modified spines of tibiotarsi I–III following the formula 0/0/4, dens dorsally with 37 chaetae	***D.betschi* Palacios-Vargas, Ferreira & Zeppelini, 2018** (Brazil)
–	Male barbulated chaetae on frontal head absent, modified spines on tibiotarsi I–III following the formula 1/1/4, dens dorsally with 46 chaetae	***D.maesorum* Palacios-Vargas, 1995** (Nicaragua)
7	Male proximal tibiotarsal organ of leg I absent	***D.lithophila* Snider, 1988** (USA)
–	Male proximal tibiotarsal organ of leg I present	**8**
8	Male’s Ant III without **c2** element (Fig. 13C), female Ant II with spiniform chaetae	***D.colombiana* Ospina & Palacios Vargas, 2009** (Colombia)
–	Male Ant III with **c2** element (Fig. 13G–I), female Ant II without spiniform chaetae	**9**
9	Eyes 6+6, unguis III without the inner tooth	***D.rhizophorae* Palacios-Vargas, Ferreira & Zeppelini, 2018** (Brazil)
–	Eyes 8+8, unguis III with the inner tooth	**10**
10	Female dorsal anal valve with 1+1 barbulated spines	***D.ramosa* (Folsom, 1932)** (Hawaii)
–	Female dorsal anal valve without barbulated spines	***D.seurati* (Denis, 1925)** (Polynesia)

* Here we did not include the species *inquirendae*. Further details of the male antennal clasper are presented in Figs 13 and 15B, C.

## ﻿Discussion

After our study, *Sphaeridia* now comprises 47 species and *Denisiella* nine recorded from the Neotropical Region, respectively (Bellinger et al. 1996–2023). *Sphaeridia* is the largest genus of Sminthurididae, but the internal relationships within this group remain unknown. Bretfeld and Gauer (1994) made an important effort dividing *Sphaeridia* into four different species groups based on the complexity of the male ventral tube. However, so far there is no clear evidence that such separation holds any phylogenetic ground. More detailed descriptions, presenting data on the chaetotaxy of the head, trunk, and appendages of both sexes could provide further data to test if the male ventral tube is a strong character to define species groups and determine the main lineages within the genus. However, many species have very brief and undetailed descriptions (see Table 1), which makes studying the internal phylogeny of *Sphaeridia* based on morphological traits difficult, and also obscuring the taxonomic comparisons among its species.

Here we separated *Denisiella* into two species groups, the taxa with and the taxa without the male nasal organ. Palacios-Vargas et al. (2018) noted that a similar, but possibly unrelated (analogous), structure is observed in males of *Arlesminthurus* Bretfeld, 1999 and some species of *Heterosminthurus* Stach, 1956 (both Bourletiellidae). In a similar way to the Neotropical *Sphaeridia*, the lack of further data concerning *Denisiella* species (see Table 2) precludes a discussion on whether the presence of the male nasal organ can be associated with other exclusive traits to better circumscribe this tentative subdivision of the genus.

## Supplementary Material

XML Treatment for Sphaeridia

XML Treatment for Sphaeridia piauiensis

XML Treatment for Denisiella

XML Treatment for Denisiella piracurucaensis

## ﻿Additional information

### Author ORCIDs

Gleyce da Silva Medeiros https://orcid.org/0000-0001-9839-2345

Clécio Danilo Dias da Silva https://orcid.org/0000-0002-7776-8830

Nerivânia Nunes Godeiro https://orcid.org/0000-0002-1669-6124

Bruno Cavalcante Bellini https://orcid.org/0000-0001-7881-9436

### Data availability

All of the data that support the findings of this study are available in the main text.
